# Anaerobic breviate protist survival in microcosms depends on microbiome metabolic function

**DOI:** 10.1093/ismejo/wraf171

**Published:** 2025-08-08

**Authors:** Karla Iveth Aguilera-Campos, Julie Boisard, Viktor Törnblom, Jon Jerlström-Hultqvist, Ada Behncké-Serra, Elena Aramendia Cotillas, Courtney Weir Stairs

**Affiliations:** Department of Biology, Lund University, Lund, 223 62, Sweden; Department of Biology, Lund University, Lund, 223 62, Sweden; Department of Biology, Lund University, Lund, 223 62, Sweden; Department of Cell and Molecular Biology, Uppsala University, Uppsala, 752 37, Sweden; Department of Biology, Lund University, Lund, 223 62, Sweden; Department of Biology, Lund University, Lund, 223 62, Sweden; Department of Biology, Lund University, Lund, 223 62, Sweden; Department of Cell and Molecular Biology, Uppsala University, Uppsala, 752 37, Sweden; Science for Life Laboratory, Department of Biology, Lund University, Lund, 223 62, Sweden

**Keywords:** protist, anaerobic metabolism, metagenomics, microbial ecology, mitochondrion-related organelles, hydrogen, syntrophy, cross-feeding

## Abstract

Anoxic and hypoxic environments serve as habitats for diverse microorganisms, including unicellular eukaryotes (protists) and prokaryotes. To thrive in low-oxygen environments, protists and prokaryotes often establish specialized metabolic cross-feeding associations, such as syntrophy, with other microorganisms. Previous studies show that the breviate protist *Lenisia limosa* engages in a mutualistic association with a denitrifying *Arcobacter* bacterium based on hydrogen exchange. Here, we investigate if the ability to form metabolic interactions is conserved in other breviates by studying five diverse breviate microcosms and their associated bacteria. We show that five laboratory microcosms of marine breviates live with multiple hydrogen-consuming prokaryotes that are predicted to have different preferences for terminal electron acceptors using genome-resolved metagenomics**.** Protist growth rates vary in response to electron acceptors depending on the make-up of the prokaryotic community. We find that the metabolic capabilities of the bacteria and not their taxonomic affiliations determine protist growth and survival and present new potential protist-interacting bacteria from the *Arcobacteraceae*, *Desulfovibrionaceae,* and *Terasakiella* lineages. This investigation uncovers potential nitrogen and sulfur cycling pathways within these bacterial populations, hinting at their roles in syntrophic interactions with the protists via hydrogen exchange.

## Introduction

Anoxic and hypoxic environments serve as habitats for a myriad of microorganisms, including unicellular eukaryotes (protists) and prokaryotes. To thrive in low-oxygen environments, protists and prokaryotes often establish specialized metabolic cross-feeding associations such as syntrophy with other microorganisms [1–3]. Some syntrophic interactions in low-oxygen environments are key to biogeochemical functions [3, 4], agriculture [5], and bioelectricity generation [6]. For example, nearly all biological methane emissions on our planet derive from syntrophic interactions between methanogenic archaea and hydrogen-producing microorganisms [7]. From an evolutionary standpoint, establishing syntrophic relationships allow collaborating organisms to persist in a new environment that could not be accessed individually and can lead to the evolution of new lineages shaped by co-evolution of each partner [3, 8].

Syntrophic interactions between anaerobic protists and bacteria have been proposed across eukaryotes within the metamonads [9], diatoms [10], discobids [11], ciliates [12–14], rhizarians [15], amoebozoans [16], dinoflagellates [17], and breviates [18]. In some of these cases, the anaerobic eukaryote hosts prokaryotic endo- or ectosymbionts that likely utilize hydrogen produced by their hosts to fuel energy conservation via methanogenesis, nitrate reduction, or sulfate reduction, yielding methane, nitrate, ammonium, or hydrogen sulfide. One of these examples includes the breviate protist *Lenisia limosa* [18].

Breviates are free-living amoeboflagellate protists that have only been identified in hypoxic or anoxic environments. They are part of the eukaryotic supergroup “Obazoa” where they branch sister to the opisthokonts (animals and fungi) and apusomonads [19]. Breviates possess specialized anaerobic mitochondria, so-called mitochondrion-related organelles (MROs), that likely couple ATP synthesis to hydrogen production rather than oxidative phosphorylation [18, 20, 21]. Attempts to establish axenic cultures have been unsuccessful, suggesting that the protist’s growth might rely on interactions with prokaryotes. Indeed, a previous study proposed that the breviate *L. limosa* engages in a mutualistic facultative association with an epibiont bacterial partner, *Arcobacter* sp. EP1 [18]. In this system, the protist likely produces hydrogen which is used by the epibiont to fuel denitrification in the presence of nitrous oxide or nitrate. It was previously proposed that *L. limosa,* like other protists and some bacteria, can perform electron confurcation whereby a low potential (e.g. ferredoxin) and a higher potential [e.g. NAD(P)H] electron donor are used *in concert* to fuel hydrogen production by a specialized [FeFe]-hydrogenase [18]. Such a reaction requires a low partial pressure of hydrogen that can be facilitated by a hydrogen-scavenging epibiont [22].

Here, we examine the diversity of breviate-associated bacteria (including *Arcobacteraceae*) and cross-feeding potential of five breviate-containing microcosms. We tested the hypothesis that different electron acceptors would influence the growth of the protists depending on the metabolic capabilities of the prokaryotic community [3, 23, 24]. Using metagenomics, fluorescent *in situ* hybridization, and amplicon sequencing analysis, we identify prokaryotes that might be important for the protist’s survival under different conditions. We find that one breviate species can only grow in the presence of sulfate whereas another species favors growth with nitrate over sulfate, suggesting that differences in the nature of the prokaryotic community (e.g. sulfate reducers or nitrate reducers) can impact the ability of the protist to survive in anoxia.

## Materials and Methods

### Microcosm establishment and maintenance

Breviate microcosms PCE, FB10N2, LRM1b, LRM2N6, and *Pygsuia biforma* were provided by Yana Eglit and Alastair Simpson [25]. Briefly, shoreline sediment was collected from Prince Edward Island, Canada (PCE), False Bay, San Juan Island, WA, USA (FB10N2), and Sant Carles de la Ràpita, Spain (LRM1b and LRM2N6). Microcosms were established by inoculating 1–2 g of sediment into 12 ml sterile natural seawater (Northwest Arm, Halifax, Nova Scotia, Canada) supplemented with 3% LB (lysogenic broth) in a 15 ml conical tube. Enrichment cultures were monitored by light microscopy every 1–2 d, and breviate-like organisms were enriched by serial dilution [25] (Supplementary Datafile S1). We amplified and sequenced the 18S ribosomal RNA (rRNA) gene using EukA and EukB primers (Supplementary material [26, 27]). Microoxic breviate cultures were maintained at 20°C in seawater (Instant Ocean, Aquarium System 216034, France) supplemented with 3% LB (LBSW), in 15 ml plastic tubes with minimal headspace and passed weekly. Anoxic cultures were maintained in glass serum flasks where headspace was replaced by argon. The differential interference contrast (DIC) images of the breviates were captured using a Axio Observer Z1 motorized inverted fluorescence microscope (Zeiss, Germany) and processed with ImageJ2 2.14.0/1.54f. DNA isolation for metagenomic sequence and amplicon sequencing are described in the Supplementary Material.

### 18S rRNA gene environmental sequencing survey and phylogenetics

The breviate 18S rRNA gene sequences from the microcosms (PCE, LRM1b, FB10N2, LRM2N6) and previously reported sequences (*P. biforma,* KC433554.1; *Breviata anathema,* AF153206.1; *Subulatomonas tetraspora,* HQ342676.1; *L. limosa,* KT023596.1) were used as queries against the Integrated Microbial Next Generation Sequencing (IMNGS) platform Version 1.0 Build 2504 [26] to retrieve sequences longer than 200 bp and >97% identical to at least one of the query sequences (Supplementary Datafile S1). The *L. limosa* query failed to retrieve any sequences from the IMNGS database. To validate the provenance of the retrieved sequences, we performed an additional taxonomic assignment of all the putative breviate sequences using SILVA 138 SINA search-and-classify [27] using a 97% cut-off and removed all non-Obazoan, Fungi, and Opisthokonta sequences. The putative breviate environmental sequences were added to a dataset of annotated breviate sequences available on Genbank and metaPR2 [28] together with a selection of apusomonad sequences as an outgroup. Sequences were aligned using SSU-align and masked using SSU-mask [29] using default settings. Phylogenies were inferred using IQTREE v2.0 [30, 31] under the best-scoring model of evolution decided by ModelFinder and 500 nonparametric bootstraps (-b 500) (Supplementary Datafile S2).

### Growth measurements with different electron acceptors in anoxia

To test which electron acceptors impact breviate growth, we grew anoxic microcosms in sulfate-free and nitrate-free 2 mM 4-(2-Hydroxyethyl)piperazine-1-ethanesulfonic acid (HEPES)-buffered (pH 7.0) defined seawater supplemented with 3% LB (dSW): dSW (no added electron acceptors), dSW-NIT (supplemented with 2 mM KNO_3_), and dSW-SULF (supplemented with 10 mM MgSO_4_), each supplemented with prey bacteria (*Klebsiella pneumoniae,* 1 × 10^9^ cells/ml). *Klebsiella pneumoniae* was a gift from Dr Elisabeth Gauger (Lund University). dSW was prepared with 27.72 g of NaCl, 0.67 g of KCl, 1.36 g of CaCl_2_.2H_2_O, 9.32 g MgCl_2_.6H_2_O, and 0.18 g NaHCO_3_ to 1 l, modified from [9] and supplemented with 30 ml of LB. Three biological replicates were prepared by inoculating 5 ml of *P. biforma* or LRM1b breviate cultures in 55 ml of dSW, dSW-NIT, or dSW-SULF in 100 ml serum flasks.

To generate the growth curves, 1 ml of each culture was collected using anoxic technique with a 22 G × 1 1/4″ needle (4710007030, HSW Henke-Ject, China) and a syringe (4020-X00V0, HSW Henke-Ject, Germany) at 0, 3, 5, 7, and 10 d and analyzed immediately using a Benchtop B3 series FlowCAM (Yokogawa Fluid Imaging Technologies Inc., Scarborough, Maine, USA), a fluid particle imaging system designed to capture and analyze particles in liquid samples. For each sample, the FlowCAM captured 1000 particles in the 3–40 μm diameter range, with three technical replicates generated per sample. All images are available at doi.org/10.17044/scilifelab.28254575.v1. To distinguish images containing a protist-like object from nonprotist material, we developed a Gradient Boosting Classifier (https://github.com/theLabUpstairs/FlowCam_Image_Classifier) by training on the image metadata variables with the most relevant features selected using Recursive Feature Elimination based on a Random Forest Classifier. The protist concentration in each sample was estimated by dividing the number of identified protist images by the volume of fluid imaged (Supplementary Datafile S4), and statistical analyses were performed using a two-way analysis of variance (ANOVA) (Supplementary Datafile S5).

### Amplicon sequencing

To explore the changes for the prokaryotic community in response to nitrate, we collected DNA from anoxic and microoxic microcosms grown in LBSW (containing sulfate) or LBSW-NIT (supplemented with 2 mM KNO3) for 7 d. The V4 region of the 16S rRNA gene was amplified from each biological replicate in three technical replicates using the barcoded primers 515F [32] and 806R [33] (Supplementary Material). Library preparation and sequencing with MiSeq System was performed by Eurofins with their NGSelect Amplicon 2nd PCR service. Adapter sequence removal and read merging were performed by Eurofins using Cutadapt v2.7 [34] and FLASH v2.2.00 [35], respectively. The resulting data were processed using qiime2 v2023.9 [36] (Supplementary Material). We calculated the relative abundance [36] and visualized the data in R, excluding the food bacteria. We calculated differential abundance across conditions with the analysis of composition of microbiomes (ANCOM) [37] specifically comparing the LBSW-NIT vs. LBSW media in anoxic atmosphere and LBSW-NIT vs. LBSW in a microoxic atmosphere.

### Metagenomic sequencing, assembly, and binning

For each microcosm, one (PCE, LRM1b and LRM2N6) or two (FB10N2) DNA sequencing libraries were prepared and sequenced by Eurofins Genomics on the NovaSeq 6000 System (Illumina; standard genomic library, S4 PE150 XP) (Supplementary Datafile S6). Read quality was assessed using fastqc 0.11.9 [38], and reads were trimmed using Trimmomatic 0.39 [39]. All strains were assembled independently using Spades 3.15.2 (options: –meta) [32]. Long-read metagenomic sequencing of microcosm DNA was performed by the National Genomics Infrastructure Sweden (PCE, LRM1b, LRM1N6, FB10N2; kit: SQK-LSK109) or in-house (*P. biforma,* kit: SQK-NBD114.24) with ONT ligation library prep with barcoding on one ONT PromethION flowcell (NBIS: FLO-PRO002 and in-house: FLO-PRO114M). Details on base-calling and adaptor trimming can be found in Supplementary Material. Sequencing reads from each microcosm were assembled independently using Flye 2.9.1 (--meta) [33]. Contig clustering, manual binning, and gene calls were performed using anvi’o 8 [34]. Scripts related to metagenome assembly and binning, metagenome-assembled genome (MAG) reassemblies, and annotation are provided on figshare and Supplementary Material. Briefly, MAGs were reassembled into high-quality genomes for *Arcobacteraceae*, *Desulfovibrionaceae,* and *Terasakiella* by mapping back the reads from each sample to each MAG using bowtie2 2.4.2 (short reads) [35], minimap2 2.24-r1122 (long reads), and samtools 1.14 (both). Reads were processed through iterative assemblies and contig clustering using Trycycler v0.5.4 to generate consensus long-read assemblies [36]. Read correction was performed using medaka v1.7.2 (https://github.com/nanoporetech/medaka; long reads for PCE, FB10N2, LRM1b, LRM2N6) or medaka v2 (*P. biforma*) and polypolish 0.5.0 (short reads for PCE, FB10N2, LRM1b, LRM2N6) [37]. Genome statistics are summarized in Supplementary Datafile S6, where the listed gene IDs correspond to locus_tag entries in the genome annotations submitted to the National Center for Biotechnology Information (NCBI) under BioProject PRJNA1084235.

### Taxonomic assignment, phylogenomic tree construction, and annotation of the assembled genomes

Taxonomic assignment of *Arcobacteraceae, Desulfovibrionaceae,* and *Terasakiella* genomes was performed using GTDBtk 2.4.0 [40]. All genomes were placed in GTDB phylogenomic tree with pplacer 1.1 [40, 41] (--classify_wf) to estimate the taxonomic placement. Taxonomic inference was refined using the --denovo_wf with defined outgroups (doi.org/10.17044/scilifelab.28254575.v1) and visualized with iTOL 7 [42]. Gene predictions were performed with prodigal through anvi’o 8 [43]; metabolic annotations were performed by searching the Kyoto Encyclopedia of Genes and Genomes (KEGG) database [44] with hmmsearch [45] through anvi’o 8.

### Fluorescence *in situ* hybridization

Breviates were incubated on a slide overnight in a moist chamber in an anoxic jar with Anaerocult A (Millipore 113 829, Sparks, Maryland, USA) and subsequently fixed in 4% formaldehyde (ThermoScientific 28 906, Rockford, IL, USA) for 15 min. Slides were rinsed with distilled water, immersed in ethanol, and incubated with hybridization buffer (Supplementary Material) containing 5 ng/μl of fluorescence *in situ* hybridization (FISH) probes targeting *Arcobacteraceae* [46], *Desulfobacterota* [9], and *Terasakiella* (Supplementary Datafile S2). After incubation, slides were washed and stained with 4',6-diamidino-2-phenylindole (DAPI). Slides were mounted with SlowFade Diamond Antifade Mountant (ThermoScientific P36970, Eugene, Oregon, USA) and imaged on a Axio Imager.z2 microscope (Zeiss, Germany), using a Plan-Apochromat 100×/1.40 Oil Ph 3 M27 immersion oil objective lens. The following filters were used: set 38 for Atto 488 (excitation, bandpass (BP) 470/40 nm; emission, BP 525/50 nm), set 31 for Atto 550 (excitation, BP 565/30 nm; emission, BP 620/60 nm), set 50 for Atto 633 (excitation, BP 640/30 nm; emission, BP 690/50 nm), and set 49 for DAPI (excitation, G 365 nm; emission, BP 445/50 nm). Images were processed using linear adjustments (e.g. brightness/contrast) in ImageJ2 2.14.0/1.54 f.

## Results and Discussion

### Breviate protists are diverse and found in low-oxygen environments

**Figure 1:**
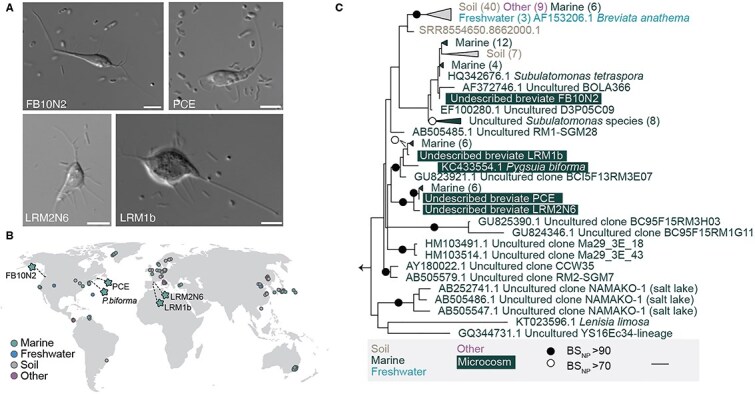
Small subunit ribosomal RNA phylogenetic analysis reveals that cultured breviates resolve into three clades within Breviatea. (A) Differential interference contrast images of the four undescribed breviate species in this study. Scale bar 5 μm. (B) Geographical locations where one or more breviate 18S rRNA gene sequence was identified in circles, breviate strain isolation sites in stars, and nature of the environment are indicated with different colours. (C) Unrooted maximum-likelihood phylogenetic analysis was computed on an alignment of 156 sequences with 1542 sites. Nonparametric bootstrap support >70% or 90% is mapped onto each bipartition with an open or closed circle, respectively. Scale bar represents 0.03 substitutions per site. Breviate sequences from microcosms are shaded. All other sequences derive from environmental sequences (colored based on sampling location) from publicly available data.

Here, we focus on five laboratory-maintained microcosms containing breviate protists (PCE, FB10N2, LRM1b, LRM2N6, or *P. biforma*) and prokaryotes isolated from brackish environments [25]. The breviate cells showed a pear-like cell shape with two flagella (usually only one is visible) emerging from the cell (Fig. 1A). All species were observed to have pronounced and sometimes branched filopodia as previously described for other breviates [18, 19, 21, 47]. We amplified and sequenced the 18S rRNA gene and used these as a query to retrieve environmental sequences >97% identical available on the IMNGS database [26]. This database houses millions of amplicon datasets of the 16S rRNA gene from public repositories including the short read archive (SRA). However, we suspected that these datasets might also contain 18S rRNA gene sequences owing to the close similarity between ribosomal RNA sequences across the tree of life. We recovered 78 putative environmental breviate sequences that derive from 68 projects targeting low-oxygen marine intertidal sediments, deep marine sediments, freshwater sediments, compost, a decommissioned mine, and soil (Fig. 1B, Supplementary Datafile S1). We reconstructed a phylogenetic tree incorporating the microcosm, IMNGS environmental sequences, and sequences annotated as breviate from the 18S rRNA gene sequence repository MetaPR2 [28]. The microcosm breviate sequences resolved into three distinct groups intermixed with environmental sequences consisting of (i) *S. tetraspora* and FB10N2, (ii) *P. biforma* and LRM1b, and (iii) PCE and LRM2N6 (Fig. 1C).

### Breviate growth rate and relative abundance of bacteria vary with availability of electron acceptors

**Figure 2:**
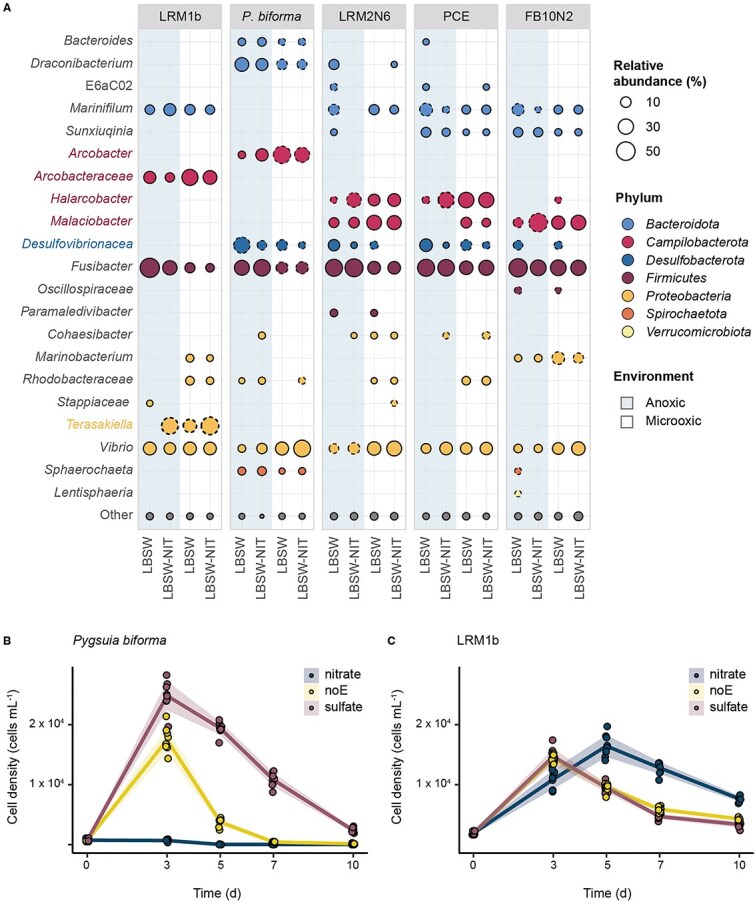
The relative abundance of bacteria in breviate microcosms varies with electron acceptors and correlates with breviate growth. (A) Bubble plot showing percentage of relative abundance (>1%) of bacteria present in each indicated microcosm (LRM1b, *P. biforma*, LRM2N6, PCE, FB10N2), growing in anoxic (shadow) or microoxic conditions (no shadow) for 7 d, in LBSW or LBSW-NIT as indicated. For visualization purposes, the relative abundance of the food bacteria is not displayed. Taxonomic assignment was made at the genus level when possible and colored based on their phyla designations. Bubbles with dashed outlines indicate taxa with statistically significant differences in relative abundance (ANCOM) between cultures within the same oxic regime (microoxia or anoxia) with or without nitrate. (B) *P. biforma* and (C) LRM1b growth curves. Breviate cultures were grown in triplicates in defined seawater (dSW), dSW-NIT, or dSW-SULF in anoxia. Protist concentration per milliliter was calculated at 0, 3, 5, 7, and 10 d. Each datapoint represents a measurement and standard deviations shown with shading. Growth rates of breviates in each microcosm cultured with different electron acceptors were compared using ANOVA and *post hoc* test, the growth rates of *P. biforma* were statistically different when grown with the different electron acceptors (*P*-value < .001), and the growth rates or LRM1b were only statistically different between dSW-NIT and dSW-SULF (*P*-value < .05), suggesting a similar growth rate between the conditions.

To understand the bacterial community associated with the breviates, we performed 16S rRNA gene amplicon analysis under microoxic and anoxic conditions supplemented with nitrate or sulfate as electron acceptors (Fig. 2A) and assessed alpha diversity, evenness, and beta diversity of the communities (Supplementary Material, Supplementary Fig. S1). *Arcobacteraceae* species were among the most abundant species in all the microcosms alongside *Vibrio* and *Fusibacter*, regardless of the presence of oxygen (Fig. 2A). In nitrate-supplemented anoxic breviate microcosms (with the exception of *P. biforma*), at least one amplicon sequence variant (ASV) displayed a statistically significantly increase in relative abundance when compared to the anoxic cultures without nitrate (Fig. 2A, Supplementary Fig. S1). Most of these organisms belong to the *Arcobacteraceae* including *Malaciobacter* (FB10N2) and *Halarcobacter* (LRM2N6 and PCE) (Fig. 2A; Supplementary Datafile S3). In the LRM1b microcosm, we detected a higher relative abundance of *Terasakiella*, and not *Arcobacteraceae*, in the presence of nitrate. In *P. biforma*, LRM2N6, PCE, and FB10N2 microcosms, the relative abundance of the taxa classified as *Desulfovibrionaceae* was higher in cultures supplemented with sulfate in comparison to the cultures supplemented with nitrate in both microoxic and anoxic microcosms (Fig. 2A; Supplementary Datafile S3). *Desulfovibrionaceae* sequences were detected in LRM1b microcosm but below the threshold for inclusion (1% relative abundance). Predictably, we observed that the addition of nitrate or sulfate under anoxia leads to changes in the relative abundance of bacteria typically associated with nitrate reduction (e.g. *Arcobacteraceae* or *Terasakiella*) or sulfate reduction (e.g. *Desulfovibrionaceae*), respectively.

To explore if there is a correlation between the bacterial community composition, protist growth, and availability of electron acceptors, we evaluated the density of *P. biforma* and LRM1b cells in anoxic conditions when supplemented with nitrate, sulfate, or no added electron acceptor over time (Fig. 2B and C). These protists were selected because they were large cells, allowing for easier visualization, and are closely related to each other but with distinct microbial communities (Fig. 2A), allowing us to test if community composition can influence protist growth. Both protists were able to grow in media with no added electron acceptors (containing only those electron acceptors present in the LB and cell inoculum). *Pygsuia biforma* grew significantly better in media with sulfate in comparison with no added electron acceptor (Fig. 2B, Supplementary Datafile S5), reaching its peak at Day 3. *Pygsuia biforma* was unable to grow in media with nitrate, suggesting that *P. biforma*’s growth is favored in the presence of active sulfate reducers and/or inhibited in the presence of active nitrate reducers. LRM1b grew better in the presence of nitrate in comparison with no added electron acceptor, reaching its peak at Day 5 (Fig. 2C, Supplementary Datafile S5). The growth of LRM1b in media with sulfate or no added electron acceptor was similar and reached its peak at Day 3, suggesting that growth of the LRM1b breviate is unaffected by the activity of sulfate reducers. However, unlike *P. biforma*, the LRM1b breviate grew in the presence of nitrate compared to sulfate-reducing conditions.

### *Arcobacteraceae*, *Desulfovibrionaceae,* and *Terasakiella* species are nitrate or sulfate reducing

**Figure 3:**
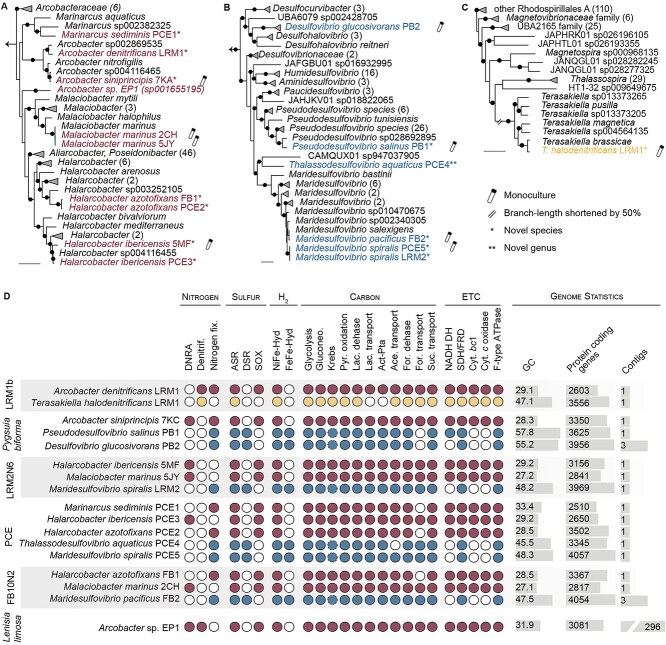
Breviate-associated bacteria resolve into three major lineages of bacteria, each with different metabolic potential. GTDB-tk computed phylogenomic trees of breviate-associated bacteria (colored) and their closest relatives (black) across (A) *Arcobacteraceae*, (B) *Desulfovibrionaceae*, and (C) *Rhodospirillales* with novel genera (**) or novel species (*) according to [48, 49]. Symbols denote monocultures (test tube icon) and branches shortened by 50% (//). Bootstrap support is indicated at key nodes (filled circles), scale bars represent 0.1 substitutions per site, and 16S rRNA gene phylogenies are shown in Supplementary Datafile S7. (D) Metabolic potential of breviate-associated bacteria. Presence (filled circles) or absence (unfilled circles) of genes and pathways for nitrogen, sulfur, and hydrogen metabolism, carbon cycling, and electron transport chains (ETCs) are shown for each species. Dotted lines indicate the pathway is incomplete. DNRA, dissimilatory nitrate reduction to ammonium; Denitrif., denitrification; Nitrogen fix., nitrogen fixation; ASR, assimilatory sulfate reduction; DSR, dissimilatory SR; Sox, sulfur oxidation; pyr., pyruvate; lac., lactate; Act-Pta, acetate kinase–phosphate acetyltransferase pathway; ace., acetate; for., formate; cyt., cytochorome; and SDH/FRD, succinate dehydrogenase/fumarate reductase. Genome statistics include GC content, number of predicted protein-coding genes, and number of contigs for each assembly.

To understand the metabolic potential of each microcosm, we used metagenomic sequencing to reconstruct genomes of their prokaryotic communities (Fig. 3). For some of the microcosms, we isolated bacteria into pure cultures and performed whole genome sequencing. From these data, we reconstructed high-quality, often circular, genomes from nine *Arcobacteraceae* (Fig. 3A)*,* six *Desulfovibrionaceae* (Fig. 3B), and one *Terasakiella* sp. (Fig. 3C and D, Supplementary Datafile S6). Nine new species names and one new genus were deposited on SeqCode [50] and classified based on the Microbial Genome Atlas guidelines (Supplementary Datafile S6 and S8) [48, 49]. Below, we discuss the metabolic potential of the community, focusing on the *Arcobacteraceae, Desulfovibrionaceae*, and *Terasakiella* members specifically related to hydrogen- and end product–sharing metabolisms. The metabolic potential of other microbes in the microcosms is discussed in the Supplementary Material.

We recovered at least one *Arcobacteraceae* MAG in each microcosm (Fig. 3A and Supplementary Datafile S7). Genes encoding hydrogen-uptake [NiFe]-hydrogenases were identified in all *Arcobacteraceae* MAGs (Fig. 3D and Supplementary Datafile S6) and are predicted to donate electrons to the quinol pool. We identified genes encoding the potential to reduce nitrate for ATP production by dissimilatory nitrate reduction to ammonium (DNRA; via NapGH, NapAB, and NrfAH) using electrons ultimately derived from hydrogen in *Halarcobacter ibericensis* PCE3 and 5MF, *Malaciobacter marinus* 2CH and 5JY, and *Arcobacter siniprincipis* 7KA (Fig. 3D). This is consistent with their increased relative abundance in media supplemented with nitrate (Fig. 2A). Unlike the *L. limosa*-associated *Arcobacter* sp. EP1, most of our breviate-associated *Arcobacteraceae* lacked the genetic capacity for complete denitrification with the exception of *A. denitrificans* LRM1 (NAP-β, NirS, NorBC, NosZ; Fig. 3D). Some of the *Arcobacteraceae* genomes encoded for nitrogen fixation via NifDKH (Fig. 3D). With respect to sulfate- and sulfide-related metabolism, each *Arcobacteraceae* MAG encoded genes related to assimilatory sulfate reduction, sulfide–quinone oxidoreductase (Sqr), and a complete sulfur oxidation (SOX) system. Collectively, these pathways suggest that the *Arcobacteraceae* are able to oxidize hydrogen sulfide, sulfite, thiosulfate, or elemental sulfur, common compounds of marine coastal environments [51–53].

Genes important for glycolysis, gluconeogenesis, the Krebs cycle, pyruvate oxidation to acetyl-CoA, acetate import (ActP) and oxidation (Ack-Pta), and lactate import (LctP) and oxidation (LldEFG, Dld; Fig. 3D) were detected in all *Arcobacteraceae*. This suggests that the *Arcobacteraceae* can use acetate or lactate as a carbon source. We also detected the potential for formate oxidation (FdhABCD), which can transfer electrons from formate to the quinol pool to fuel DNRA like other *Arcobacter* [54], although not all the strains encode for a formate transporter. The majority of the *Arcobacteraceae* genomes encoded a tripartite ATP-independent periplasmic transporter (TRAP) type or DcuAB system that is predicted to transport succinate, fumarate, and similar molecules. Succinate could serve as an electron or carbon source by the bacteria in the microcosms; in fact, *M. marinus*, *H. ibericensis,* and *A. siniprincipis* isolates can grow on succinate as the sole carbon source (Aguilera-Campos *et al*. in preparation). All the genes for aerobic respiration were found in the *Arcobacteraceae* genomes, including NADH:quinone oxidoreductase, fumarate reductase, cytochrome bc1 complex, cytochrome *c* oxidase (cbb3-type), and F-type ATPase, in agreement with their microaerophilic lifestyle.

We assembled *Desulfovibrionaceae* genomes from the *P. biforma*, FB10N2, PCE, and LRM2N6 metagenomes some of which derive from new species within the *Maridesulfovibrio* and *Pseudodesulfovibrio* genera and a new genus we named *Thalassodesulfovibrio* (Fig. 3B, Supplementary Datafile S8). All *Desulfovibrionaceae* genomes encoded for hydrogen-uptake [NiFe]-hydrogenases and formate dehydrogenases that are predicted to transfer electrons to a type-I cytochrome *c*_3_ [55, 56]. These electrons likely feed into the dissimilatory sulfate reduction (DSR) pathway via the quinone reductase (QrcABCD) and modifying (QmoABC) complexes, adenylylsulfate reductase (AprAB), and dissimilatory sulfate reductase (DsrABCD, DsrMKJOP; Fig. 3D and Supplementary Datafile S6) [55, 57, 58]. Given their gene content and increased relative abundance in microcosms supplemented with sulfate (Fig. 2C), we suspect all the breviate-associated *Desulfovibrionaceae* are sulfate-reducing bacteria (SRB). We also detected the potential for nitrogen fixation in all the *Desulfovibrionaceae* genomes, a feature of other marine SRB [59].

With respect to carbon metabolism, all of the *Desulfovibrionaceae* genomes encoded genes for glycolysis, gluconeogenesis, pyruvate oxidation, Ack-Pta and acetyl-CoA synthetase (ACS) pathways, and an F-type ATPase. They encoded a truncated Krebs cycle (lacking malate dehydrogenase, aconitate hydratase, and succinyl-CoA synthetase) and lacked aerobic respiration, similar to other *Desulfovibrionaceae* [60, 61]. All breviate-associated *Desulfovibrionaceae* genomes encoded for lactate import (LutP) and oxidation (LDH). This suggests that the *Desulfovibrionaceae* could accept electrons from either the hydrogen-uptake machinery above or lactate to fuel DSR. All *Desulfovibrionaceae* genomes encoded a TRAP-type transport system (for succinate/fumarate transport) and a fumarate reductase (FrdABC), which can likely oxidize quinols.

We only recovered a *Terasakiella* MAG (complemented with an isolate genome) in the LRM1b microcosm and this likely represents a new species, *Terasakiella halodenitrificans* (Fig. 3C, Supplementary Datafile S7). The *T. halodenitrificans* genome encodes a hydrogen-uptake [NiFe]-hydrogenase that is predicted to pass electrons via the quinol pool to the cytochrome *bc*1 complex, ultimately yielding reduced cytochrome *c* [62]. We also detected genes for Nap-mediated complete denitrification (via NapAB, NapCDH, NirS, NorBC, and NosZ; Fig. 3D, Supplementary Datafile S6) and observed an increase in the relative abundance of the *T. halodenitrificans* ASV in nitrate-supplemented microcosms (Fig. 2C). *Terasakiella halodenitrificans* encoded all the genes for glycolysis, gluconeogenesis, the Krebs cycle, formate transport, lactate dehydrogenase, and formate dehydrogenase; however, the electron carriers for these systems are unknown. *Terasakiella halodenitrificans* can likely import and metabolize acetate via an ActP and ACS, respectively. We also detected a succinate TRAP-transporter and a succinate dehydrogenase that is predicted to funnel electrons to the quinone pool. Collectively, this suggests that *T. halodenitrificans* is a facultative anaerobe that can use succinate and acetate as carbon sources.

### *Arcobacteraceae*, *Desulfovibrionaceae,* and *Terasakiella* bacteria are associated with breviate cells

**Figure 4:**
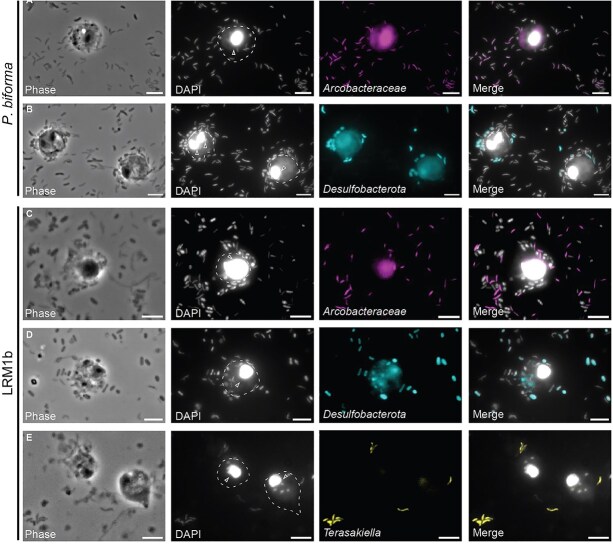
Fluorescence *in situ* hybridization (FISH) suggests a non-obligate direct interaction of *P. biforma* and LRM1b with hydrogen-consuming bacteria. *Pygsuia biforma* (A and B) and LRM1b (C–E) cultures were incubated on a slide overnight in an anaerobic glove box. Cultures were fixed (4% formaldehyde) and hybridized with 16S rRNA probes: Arc1430-Atto 488 and Arc94-Atto 488 targeting *Arcobacteraceae* cells (third panel , A and C), Delta495a-Atto 550 probe targeting *Desulfobacterota* (formerly “Deltaproteobacteria”; third panel, B and D), and Tera537-Atto 550 probe targeting *Terasakiella halodenitrificans* LRM1 (third panel, E). Cell body (outlines) and nuclei (arrow) of the protists are shown in the DAPI panel. Panels left to right: Phase, DNA stained with DAPI (the bigger circles correspond to the breviate nucleus), specific FISH probes, and merge channel of DAPI with FISH probes. Scale bar 5 μm.

To visualize if bacteria were directly interacting with the breviates, we performed FISH using probes directed against the 16S rRNA of *Arcobacteraceae*, *Desulfobacterota* (formerly “deltaproteobacteria”*)*, and *Terasakiella halodenitrificans*. *Arcobacteraceae*-positive cells are associated with protist cells at varying frequencies: PCE (70.6%), FB10N2 (32.3%), LRM1b (47.8%), LRM2N6 (12.5%), and *P. biforma* (78.3%) (Fig. 4, Supplementary Fig. S2, Supplementary Datafile S2). However, we also observed a signal for bacteria not associated with the protist (e.g. Fig. 4C). It could be that under anoxic conditions, microaerophiles such as *Arcobacteraceae* are not constitutively associated with the protist cells if they are seeking oxygen.

*Desulfobacterota*-positive cells were found to be frequently associated with protist cells in the presence of sulfate (PCE, 52.4%; FB10N2, 100%; LRM1b, 90.7%; LRM2N6, 78.6%; *P. biforma*, 78.1%). For those microcosms that contained multiple *Desulfobacterota* species, we designed probes to target specific genera of *Maridesulfovibrio* (MD1, MD2, Supplementary Fig. S3) and *Pseudodesulfovibrio* (PD1, Supplementary Fig. S4). *Maridesulfovibrio* cells were found to be associated with LRM2N6 cells (57%) with fewer associations with FB10N2 (20%) (Supplementary Fig. S3, Supplementary Datafile S2). *Pseudodesulfovibrio* did not associate with *P. biforma* cells in all examined cases (Supplementary Fig. S4, Supplementary Datafile S2). We therefore suspect that other species (e.g. *Desulfovibrio glucosivorans* PB2 or *Thalassodesulfovibrio aquaticus* PCE4) that react positively to the *Desulfobacterota* probe could be interacting with the protists. The *Terasakiella*-targeted FISH probe labeled bacterial cells in 57% of LRM1b cells grown with nitrate (Fig. 4E), compared to 10% of cells grown in the absence of nitrate (Supplementary Datafile S2).

Extracellular relationships between prokaryotes and protists can vary in the nature of the interaction. For example, some protists have sophisticated cellular structures that coordinate their prokaryotic partners [9, 11, 18, 63] and at least one micrograph shows a pilus-like structure formed between *Arcobacter* sp. EP1 and *L. limosa* [18]. However, some extracellular interactions of *Arcobacter* with other eukaryotes are transient [64]. For example, *Arcobacter* species are associated with *Osedax* worms during specific life stages. It is difficult to know if the potential association of the bacteria with the breviates is due to an interaction or just a coincidence. In the *L. limosa* study, there is no discussion or representation of non protist-associated *Arcobacter* [18]. Future experiments should interrogate the contact dependence and environmental conditions that promote breviate:prokaryote interactions, specifically to examine the function of the chemotaxis and cell:cell adhesion machinery (Supplementary Material).

### Breviates likely produce small organic acids that can be used by *Arcobacteraceae, Desulfovibrionaceae,* and *Terasakiella*

**Figure 5:**
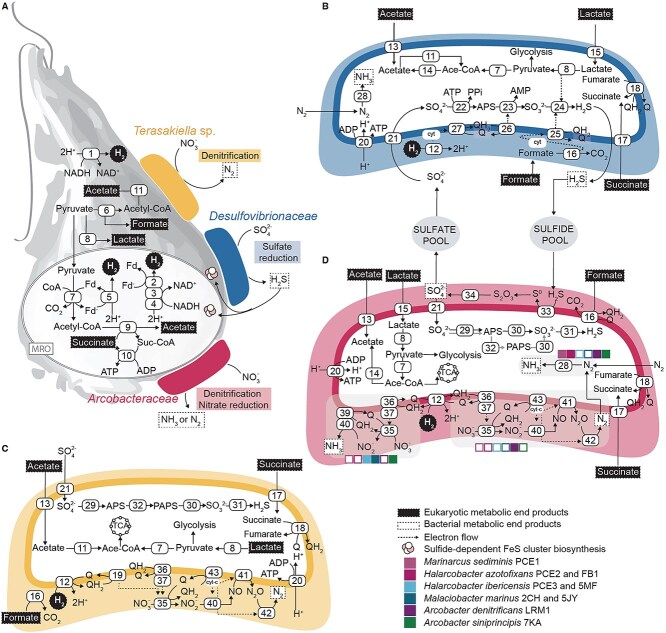
Prediction of potential metabolic cross-feeding of breviate-associated bacteria with breviates. (A) Schematic representation of a potential metabolic interaction of the breviate cell with the associated bacteria, *Desulfovibrionaceae* , *Arcobacteraceae* , and *Terasakiella* sp. The breviate cell produces acetate, succinate, lactate, formate, and hydrogen as end products (shadowed boxes) of pyruvate and energy metabolism in the cytoplasm and/or in the mitochondrion-related organelle (MRO); these products can then be used by the associated bacteria, *i.e.,* for dissimilatory sulfate reduction, dissimilatory nitrate reduction, or denitrification. Breviate metabolism was predicted from transcriptomes of the *P. biforma* [20] and *L. limosa* [18]. Predicted metabolism of *Desulfovibrionaceae* (B), *Terasakiella* sp. (C), and *Arcobacteraceae* (D) highlighting hydrogen uptake, nitrate, and sulfate metabolic pathways. Proposed end products of metabolism are shown in boxes. (1) NAD(P)H-dependent [FeFe]-hydrogenase, (2) [FeFe]-Hydrogenase, (3) NuoE: NADH:ubiquinone oxidoreductase subunit E, (4) NuoF: NADH:ubiquinone oxidoreductase subunit F, (5) Ferredoxin (Fd)-dependent Hydrogenase, (6) PFL: pyruvate formate-lyase, (7) PFO/POR: pyruvate:Fd oxidoreductase, (8) LDH: lactate dehydrogenase, (9) ASCT: acetate:succinate CoA-transferase, (10) SCS: succinyl-CoA synthetase, (11) ACS: acetyl-CoA synthetase, (12) [NiFe]-hydrogenase, (13) acetate transporter (e.g. ActP), (14) Ack/Pta: acetate kinase phosphate acetyltransferase pathway, (15) LutP: lactate permease, (16) FDH: formate dehydrogenase, (17) TRAP: tripartite ATP-independent periplasmic transporter, (18) FRD: fumarate reductase/SDH succinate dehydrogenase, (19) NapC: quinole dehydrogenase, (20) ATP synthase, (21) SulP: sulfate permease, (22) Sat: sulfate adenylyltransferase, (23) AprAB: adenylylsulfate reductase subunit A and B, (24) DsrABCD: dissimilatory sulfite reductase subunits, (25) DsrMKJOP dissimilatory sulfite reductase subunits, (26) qmoABC: quinone-modifying oxidoreductase, (27) QrcABCD: menaquinone reductase, (28) NifDKH: nitrogenase, (29) CysN: sulfate adenylyltransferase subunit 1, (30) CysH: phosphoadenosine phosphosulfate reductase, (31) CysJI: sulfite reductase, (32) CysC: adenylylsulfate kinase, (33) Sqr: sulfide:quinone oxidoreductase, (34) SOX: sulfide oxidation system, (35) NapAB: nitrate reductase, (36) NapH: menaquinone dehydrogenase, (37) NapG: menaquinone dehydrogenase, (38) NrfA: nitrite reductase, (39) NrfH: cytochrome *c* nitrite reductase, (40) NirS: nitrite reductase, (41) NorBC: nitric oxide reductase, (42) NosZ: nitrous oxide reductase, (43) Cyt bc1: cytochrome *bc*1 complex. Other abbreviations: TCA: tricarboxylic acid cycle, cyt-c: cytochrome c, Ace-CoA: Acetyl coenzyme A, Suc-CoA: succinyl-CoA.

To examine if the protists can produce metabolites compatible with the predicted metabolism of the bacteria, we queried the genome and transcriptome data of *P. biforma* and *L. limosa* as proxies for breviate metabolism to identify genes related to formate, acetate, lactate, and succinate production (Fig. 5A, Supplementary Datafile S6). *Pygsuia biforma* and *L. limosa* encode a pyruvate formate lyase and formate transporter (FocA) protein, suggesting that the protist might be capable of producing and transporting formate. Both protists are predicted to produce lactate (LDH), acetate (acetate:succinate CoA transferase), and succinate (fumarate-reducing complex II [19, 65] and succinyl CoA synthetase), although specific export transporters could not be confidently identified.

These end products are compatible with the examined bacteria and are consistent with known growth dependencies of related species. For example, most of the breviate-associated *Desulfovibrionaceae* genomes encode genes for lactate, formate, and succinate/fumarate transport and utilization (Fig. 3D). Previous studies have shown that related species (e.g. *Desulfovibrio glucosivorans, D. desulfuricans*), can grow on media supplemented with lactate, hydrogen/acetate, or formate [66, 67]. Therefore, we suspect that the breviate-associated *Desulfovibrionaceae* can likely use community-derived lactate, formate, and acetate/hydrogen as substrates for growth (Fig. 5B). Similarly, the protist-associated *Arcobacteraceae* and *Terasakiella* can likely take up lactate, acetate, and succinate (Figs 3D and 5D) using formate or hydrogen as electron donors as previously reported in other *Arcobacteraceae* [17, 61, 68] and *Terasakiella* [89, 90].

### Metabolic inferences suggest breviates can tolerate end products of community members

Previous studies in *P. biforma* and *L. limosa* predict that breviates produce hydrogen as an end product of metabolism by the action of confurcating NAD(P)H-dependent [FeFe]-hydrogenases that likely require a low-partial pressure of hydrogen to function [18, 20] (Fig. 5A). Therefore, the metabolism of the protist would benefit a hydrogen-scavenging prokaryote, which could therefore serve as a hydrogen sink to keep the partial pressure of hydrogen low as previously proposed in ciliates [69], metamonads [9, 61, 70], amoebozoans [16], and *L. limosa* [18]. Here, we propose that nitrate-reducing, denitrifying, or sulfate-reducing bacteria could serve as the hydrogen sink in the breviate microcosms (Fig. 5). For example, the LRM1b-associated *T. halodenitrificans* and *Arcobacter denitrificans* may couple hydrogen oxidation with denitrification (Fig. 5D). Similarly, the breviate-associated *Arcobacteraceae* (*H. ibericensis*, *M. marinus,* and *A. siniprincipis*) might use hydrogen as an electron donor for DNRA (Fig. 5D), as was observed for other *Campylobacterota* isolates [71]. Finally, the *Desulfovibrionaceae* might serve as the hydrogen sink via DSR (Fig. 5B) as has been proposed for other symbionts of protists [61]. These microcosm inferences might reflect the ecology of these organisms in their natural environments as sequences from breviates recovered from environments known to host denitrifying and sulfate-reducing bacteria (e.g. Marmara sea sediment [66]: HM103514, HM103491; Disko island tidal sediments [72]: EF100226, EF100280, EF100389, EF100391, EF100407, EF100410).

In contrast to *L. limosa*, four of our microcosms lack bacteria capable of complete denitrification and *P. biforma* was unable to grow in the presence of nitrate. We suggest that the denitrification potential of the most abundant prokaryote in the LRM1b microcosm, *T. halodenitrificans* (and less abundant *A. denitrificans*) explains why LRM1b and not *P. biforma* can grow with nitrate. This suggests that, in general, denitrification is compatible with LRM1b survival or that the byproducts of dissimilatory nitrate reduction (e.g. ammonia) can be toxic to *P. biforma*, and not *L. limosa* or LRM1b microcosms. If nitrate is available, then nitrate-reducing or denitrifying bacteria (*Arcobacteraceae*, *Terasakiella*) will use LRM1b microcosm end products and outcompete the sulfate-reducing bacteria due to more favorable energy potential of DNRA and denitrification over DSR [73, 74]. If sulfate is available, sulfate-reducing *Desulfovibrionaceae* and sulfide-oxidizing *Arcobacteraceae* species can use microcosm end products and likely maintain sulfide homeostasis compatible with *P. biforma* growth.

Although sulfide can be toxic to eukaryotes [75–77], we suspect that the breviates might rely on sulfide for biosynthetic purposes. Most eukaryotes synthesize Fe-S clusters using the iron–sulfur cluster (ISC) system that relies on cysteine-derived sulfide. *Pygsuia biforma* lacks the ISC system and instead encodes an archaeal minimal sulfur mobilization system [20] that, in archaea, relies on sulfide [78]. Therefore, we suspect that the breviates might be able to directly utilize environmental sulfide for Fe-S cluster metabolisms and have increased resilience in sulfidic environments such as deep sea [66] or tidal [72] sediments. In addition, sulfide homeostasis in the microcosms could be regulated by sulfide-oxidizing *Arcobacteraceae* via their SOX system (Fig. 5D) as seen in marine invertebrate symbioses involving *Arcobacteraceae* [64] and other bacteria [79–81].

### Limited evidence for genome reduction or co-evolution of the breviate protists and their associated bacteria

The nature of known eukaryote:prokaryote interactions is diverse, ranging from transient associations to obligate symbioses [82], and is influenced by the environment, as well as the metabolic needs of the interacting partners. For example, in some anaerobic ciliates, the metabolic activities of symbiotic methanogenic archaea or sulfate-reducing bacteria boost the ciliate’s metabolic efficiency [83]. Over evolutionary time, symbiotic interactions can lead to co-evolution of both host and symbiont, including the reduction of symbiont genomes [12, 84], mutual dependence [16], and even vertical transmission or replacement of symbionts across speciation events [85, 86]. To detect signatures of genome reduction, we surveyed the amino acid biosynthetic capabilities and pseudogene content in the breviate-associated bacteria. We found that biosynthetic capabilities and the number of pseudogenes varied across the *Arcobacteraceae* or *Desulfovibrionaceae* genomes independent of their presumed symbiotic lifestyles (Supplementary Discussion, Supplementary File S6). We failed to detect significant co-diversification patterns between the species trees for the protists and *Arcobacteraceae* or *Desulfovibrionaceae* based on a Mantel test or Procrustes Application to Cophylogenetic Analysis (Supplementary Material). With present data and methods, we cannot determine if failure to detect co-evolution is due to the diverse relationships among the breviate species (22% variation in identity at the 18S rRNA gene sequence) and narrow sampling of the lineage compared to well-studied host:symbiont systems [87].

The diverse nature of the *Desulfovibrionaceae* and *Arcobacteraceae* across phylogenomic trees (Fig. 3A) suggests that their co-occurrence with breviates may not be the result of co-speciation but rather a propensity or metabolic capabilities for these lineages to engage in interactions with eukaryotes. Indeed, *Arcobacteraceae* species have been shown to associate with other eukaryotes as extracellular epibionts or ectosymbionts [18, 64, 88], and in some cases, these associations are transient [64]. *Desulfovibrionaceae* are also metabolic partners of protists [61, 70]. Even *Terasakiella* species, like *T. pusilla* (known as *Oceanospirillum pusillum* or *Spirillum pusillum)*, was isolated from a marine shellfish [65], and other species from the *Rhodospirillales* order have been found in association with other eukaryotes [67, 68].

Here, we present the microbial complement of five laboratory-maintained protist microcosms. We demonstrate that protist growth is influenced by the metabolism of the bacteria in the consortium based on the availability of different electron acceptors like sulfate and nitrate. Our findings corroborate previous observations of the *Arcobacter:Lenisia* microcosm and expand this association to five diverse breviate species, identifying *Desulfovibrionaceae* and *Terasakiella* as new potential partners. Several questions remain about the metabolic interactions between anaerobic breviates and bacteria with respect to the contact dependence and metabolic nature of these interactions. By increasing the sampling of known breviate diversity, it is evident that, much like *L. limosa*, breviate anaerobic growth is influenced by its surrounding prokaryotic community. These interactions likely depend on the metabolism of the bacterial community and availability of electron acceptors and not necessarily on the taxonomic affiliation of the bacteria. Future research should focus on how these organisms might interact in the natural environment and whether this relationship has evolved into a specialized contact-dependent interaction and/or symbiosis. Furthermore, distinct pathways of adaptation to anoxia in endobiotic, marine, and freshwater environments, where predation, competition, and nutrient availability vary, might also play a role.

## Supplementary Material

Supplementary_Figure_S1_wraf171

Supplementary_Figure_S2_wraf171

Supplementary_Figure_S3_wraf171

Supplementary_Figure_S4_wraf171

SupplementaryMaterial_R2_noTC_250721_wraf171

Supplementary_DataFileS1_18S_geography_wraf171

Supplementary_DataFileS2_PCR_FISH_PHYLOGENY_R1_wraf171

Supplementary_DataFileS3_AmpliconQIIMEAnalysis_R1_wraf171

Supplementary_DataFileS4_growthcurve_Pygsuia_LRM1b_wraf171

Supplementary_DataFileS5_anova_growth_rates_wraf171

Supplementary_DataFileS6_GenomeInformation_R1_wraf171

Supplementary_DataFileS7_SSUtrees_R1_wraf171

Supplementary_DataFileS8_seqcode_wraf171

## Data Availability

Amplicon reads, metagenomic reads, and assembled genomes of the prokaryotes in the breviate microcosm have been deposited to NCBI in BioProject PRJNA1084235. Descriptions of the datasets are available in Supplementary Datafiles S3 and S6. Breviate 18S rRNA gene sequences have been deposited under the Genbank Accessions PV036398 (PCE), PV036399 (FB10N2), PV036400 (LRM1b), and PV036401 (LRM2N6). Metagenome assemblies and metagenome-assembled genomes, gene calls, taxonomic classification, metabolic annotation, scripts, quality assessment, raw microscopy pictures, and flowCAM data can be found in figshare (doi.org/10.17044/scilifelab.28254575.v1). Scripts for the flowCAM data classifier can be found https://github.com/theLabUpstairs/FlowCam_Image_Classifier.
