# Supplementary figures and images for: Anaerobic breviate protist survival in microcosms depends on microbiome metabolic function
Source: ISME J. 2025 Aug 8;19(1):wraf171. doi: 10.1093/ismejo/wraf171 (PMC12453579; doi:10.1093/ismejo/wraf171)

A

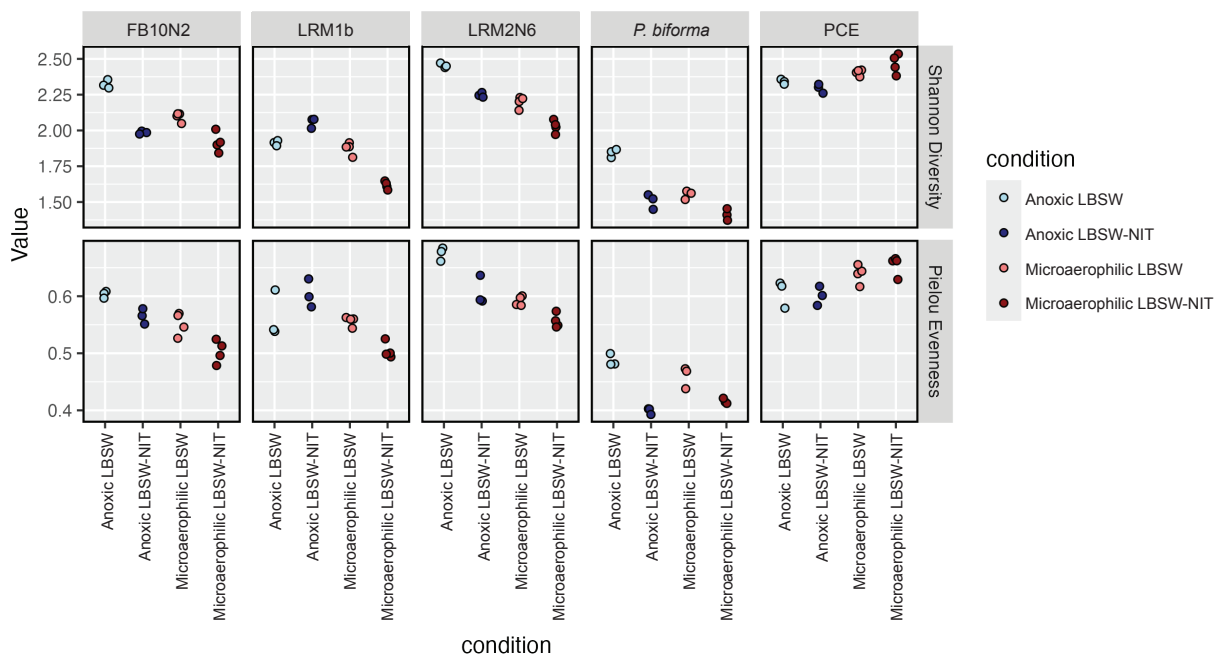

B

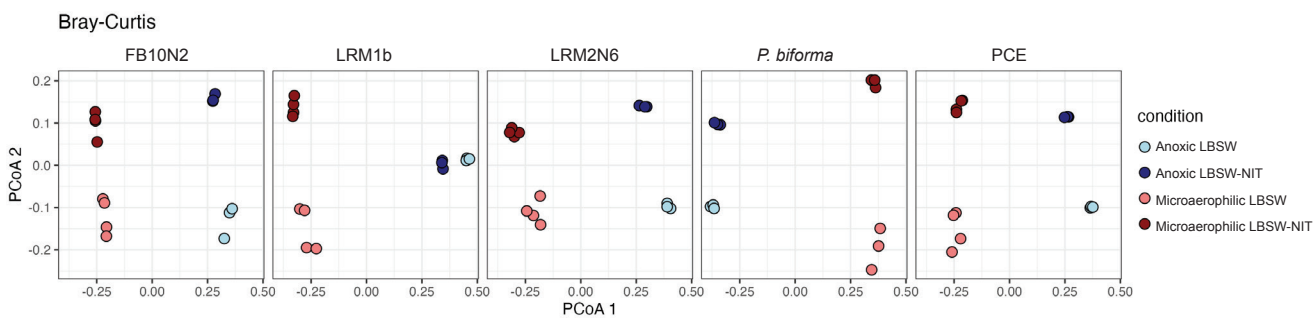

Supplement: Supplementary_Figure_S1_wraf171 [file supplementary_figure_s1_wraf171.pdf]

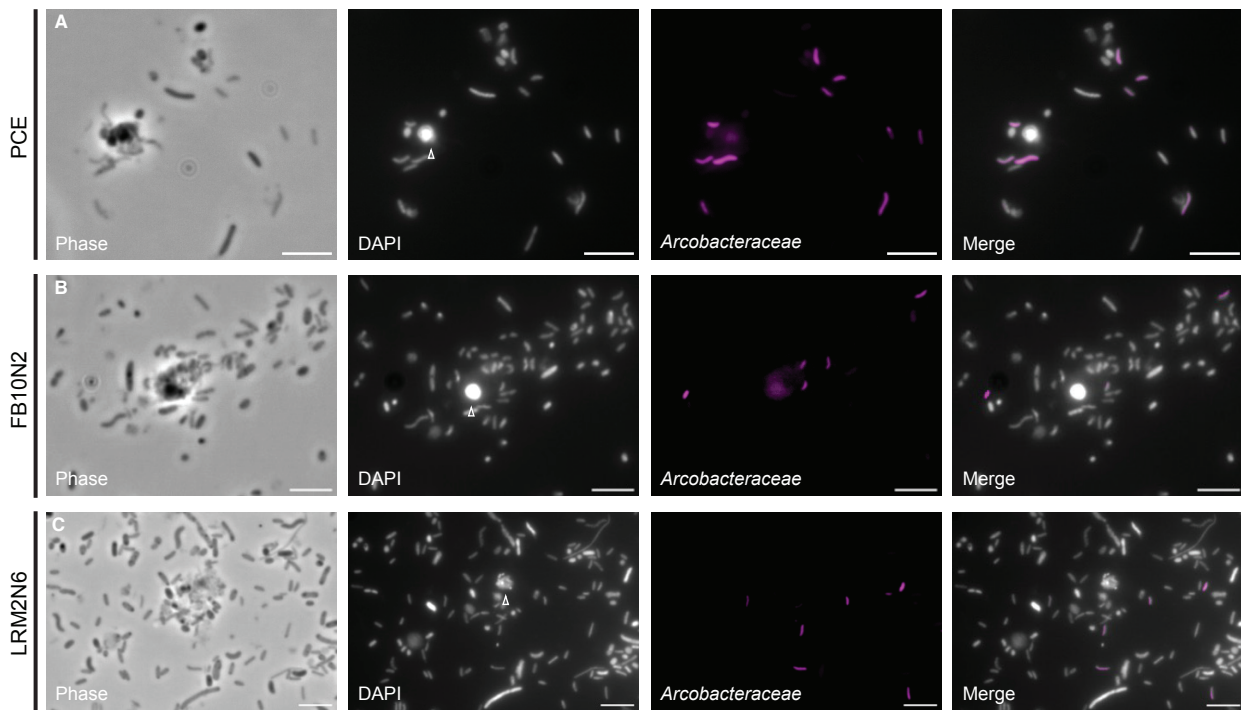

Supplement: Supplementary_Figure_S2_wraf171 [file supplementary_figure_s2_wraf171.pdf]

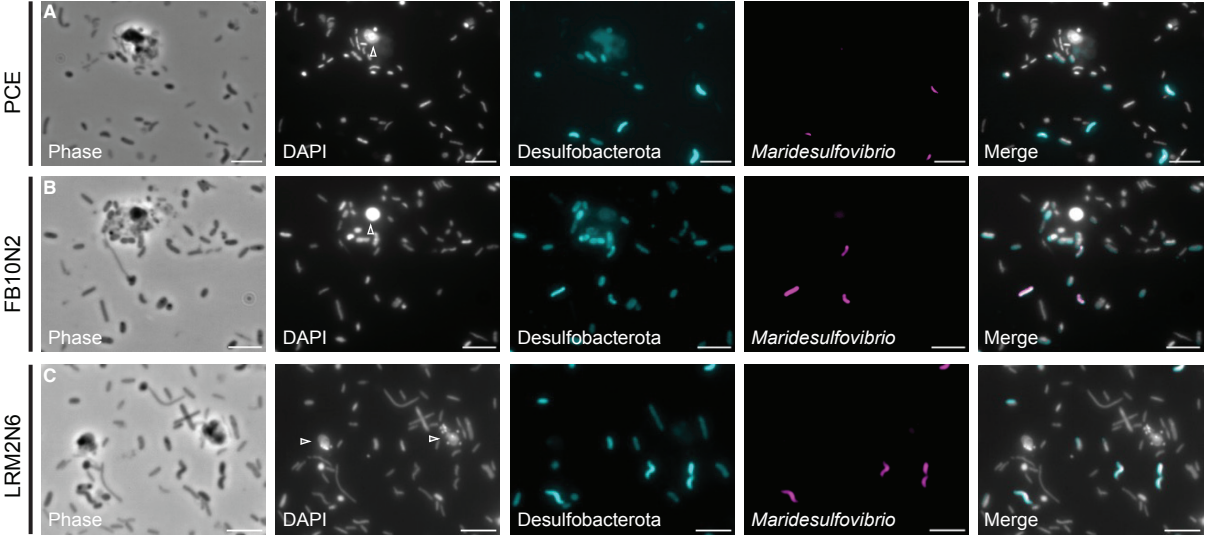

Supplement: Supplementary_Figure_S3_wraf171 [file supplementary_figure_s3_wraf171.pdf]

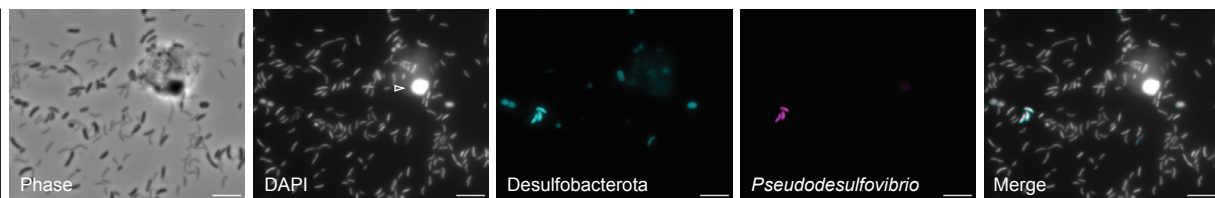

Supplement: Supplementary_Figure_S4_wraf171 [file supplementary_figure_s4_wraf171.pdf]
