# Supplementary material for: Anaerobic breviate protist survival in microcosms depends on microbiome metabolic function
Source: ISME J. 2025 Aug 8;19(1):wraf171. doi: 10.1093/ismejo/wraf171 (PMC12453579; doi:10.1093/ismejo/wraf171)
Supplement: SupplementaryMaterial_R2_noTC_250721_wraf171 [file supplementarymaterial_r2_notc_250721_wraf171.docx]

**SUPPLEMENTARY MATERIAL FOR AGUILERA-CAMPOS, BOISARD ET AL.**

**Anaerobic breviate protist survival in microcosms depends on microbiome metabolic function.**

Karla Iveth Aguilera-Campos^1^*, Julie Boisard^1^*, Viktor Törnblom^1^, Jon Jerlström-Hultqvist^2^, Ada Behncké-Serra^1^, Elena Aramendia Cotillas^1^, Courtney Weir Stairs^1,2,3^*These authors contributed equally to the work

**AFFILIATIONS**
 ^1^Department of Biology, Lund University, Lund, Sweden ^2^Department of Cell and Molecular Biology, Uppsala University, Uppsala, Sweden
^3^Science for Life Laboratory, Department of Biology, Lund University, Lund, Sweden

**CORRESPONDING AUTHOR**
Email: [courtney.stairs@biol.lu.se](mailto:courtney.stairs@biol.lu.se); [courtney.stairs@icm.uu.se](mailto:courtney.stairs@icm.uu.se)

Mailing address:

Courtney Stairs
Molecular Biosciences, Department of Biology
Sölvegatan 35
Lund, Sweden
223 62

Courtney Stairs
Molecular Evolution, Department of Cell and Molecular Biology
Husargatan 3
Uppsala, Sweden
752 37

**COMPETING INTERESTS**

The authors declare no competing financial interests.

# SHORT TITLE

H_2_-sharing between

**Table of contents**

[SUPPLEMENTARY MATERIALS AND METHODS 4](#_Toc204007534)

[DNA isolation and preparation for metagenomic sequencing 4](#_Toc204007535)

[18S rRNA gene amplification and sequencing 4](#_Toc204007536)

[Amplicon sequencing 5](#_Toc204007537)

[Amplicon sequencing diversity analysis 6](#_Toc204007538)

[Using DiSCo to predict dissimilatory sulfate reduction metabolism 7](#_Toc204007539)

[Detection of denitrification associated proteins 7](#_Toc204007540)

[Metagenomic sequencing, assembly and binning 7](#_Toc204007541)

[Bacterial isolation and genome sequencing 8](#_Toc204007542)

[16S rRNA phylogenetic trees 9](#_Toc204007543)

[Average Nucleotide Identity (ANI) comparisons between genomes 9](#_Toc204007544)

[Detecting signs of co-evolution 10](#_Toc204007545)

[Pseudogene detection 10](#_Toc204007546)

[GapMind for prediction of amino acid biosynthesis 11](#_Toc204007547)

[Fluorescence *in situ* hybridization 11](#_Toc204007548)

[SUPPLEMENTARY RESULTS & DISCUSSION 13](#_Toc204007549)

[All breviate associated bacteria encode genes for chemosensory and flagellar machinery 13](#_Toc204007550)

[Limited evidence for co-diversification of bacteria and breviates in the microcosms 13](#_Toc204007551)

[Exploring amino acid biosynthetic capabilities and pseudogenization in breviate-associated bacteria 14](#_Toc204007552)

[Additional bacterial candidates that can interact with the breviates 15](#_Toc204007553)

[SUPPLEMENTARY FIGURES & TABLES 17](#_Toc204007554)

[Supplementary figure S1: Differences in alpha and beta diversity of the bacterial community in the breviate microcosm. 18](#_Toc204007555)

[Supplementary figure S2: Fluorescence *in situ* hybridization (FISH) suggests a direct interaction of PCE, FB10N2 and LRM2N6 with *Arcobacteraceae*. 19](#_Toc204007556)

[Supplementary figure S3: Fluorescence *in situ* hybridization (FISH) suggests a non-direct interaction of PCE, FB10N2 and LRM2N6 with *Maridesulfovibrio* species. 20](#_Toc204007557)

[Supplementary figure S4: Fluorescence *in situ* hybridization (FISH) suggests a non-direct interaction of *P. biforma* with *Pseudodesulfovibrio*. 21](#_Toc204007558)

[DESCRIPTION OF SUPPLEMENTARY DATA FILES 22](#_Toc204007559)

[Supplementary Datafile S1 (.xlsx) 22](#_Toc204007560)

[Supplementary Datafile S2 (.xlsx) 22](#_Toc204007561)

[Supplementary Datafile S3 (.xlsx) 22](#_Toc204007562)

[Supplementary Datafile S4 (.xlsx) 22](#_Toc204007563)

[Supplementary Datafile S5 (.html) 22](#_Toc204007564)

[Supplementary Datafile S6 (.xlsx) 22](#_Toc204007565)

[Supplementary Datafile S7 (.pdf) 23](#_Toc204007566)

[Supplementary Datafile S8 (.pdf) 23](#_Toc204007567)

[Figshare contents 23](#_Toc204007568)

[REFERENCES 24](#_Toc204007569)

# SUPPLEMENTARY MATERIALS AND METHODS

## DNA isolation and preparation for metagenomic sequencing

Cells were collected by centrifugation at 2500 x g for 5 min, and the cell pellet was resuspended in 500 μl of extraction buffer (10 mM Tris-HCl pH 8.0, 10 mM EDTA pH 8.0 and 2% SDS). Proteinase K (20 mg/ml) (ThermoScientific AM2542, Germany) was added to the solution to a final concentration of 770 μg/ml. The tubes were mixed by inversion and incubated at 56°C for 3 h. The DNA was extracted by standard phenol:chloroform extraction using equal volume phenol: chloroform: isoamyl alcohol (ThermoScientific 327111000, Israel) followed by a chloroform extraction to remove residual phenol. The DNA was precipitated with 1/10 volume of 3 M sodium acetate buffer solution pH 5.2 (ThermoScientific R1181, Lithuania) followed by the addition of 2.5 volumes of ice-cold absolute ethanol. The samples were mixed and stored at -20° overnight. To recover the DNA, the samples were collected by centrifugation at 13000 x g for 30 min at 4°C and the resulting pellet was washed with 70% ethanol twice by collection at 13000 x g for 30 min at 4°C and ultimately resuspended in 10 mM Tris buffer, pH 8.0. DNA for metagenome sequence was isolated from PCE, FB10N2, LRM1b, LRM2N6 and *Pygsuia biforma* breviate cultures, cultured in 50 ml conical tubes with 45 ml of LBSW at room temperature for seven days (microaerophilic). For nanopore sequencing, the DNA was treated with 1 mg/ml RNase A (ThermoScientific LT-02241, Lithuania), followed by incubation at room temperature for 10 min. The DNA was quantified with Qubit dsDNA HS assay kit (Invitrogen Q32854, Eugene, Oregon, USA).

## 18S rRNA gene amplification and sequencing

To recover the 18S rRNA gene sequences of the undescribed breviates, we isolated the DNA from PCE, FB10N2, LRM1b and LRM2N6 breviate cultures as described above. The 18S rRNA gene was amplified from 1 ng of DNA using eukaryote-specific primers EukA and EukB[1] and GoTaq G2 Green Master Mix (Promega M7823, Madison, WI, USA). The thermocycler conditions are outlined in Supplementary Datafile S3. PCR products were separated on a 1% agarose gel, and the products of ~1800 bp were purified using the Nucleospin Gel and PCR clean-up (Macherey-Nagel 740609.50, Germany). Fragments were cloned into the pGEM-T easy vector systems (Promega A3600, Madison, WI, USA) and transformed into chemically competent DH5ɑ *Escherichia coli* chemical competent cells using standard methods[2]. Plasmids were isolated from positive clones using Nucleospin plasmid purification (Macherey-Nagel 740588.50, Germany) and sequenced using Sanger sequencing (Eurofins).

## Amplicon sequencing

To explore the changes for the prokaryotic community in response to nitrate under microaerophilic conditions, four biological replicates of each breviate microcosm were grown in LBSW or LBSW-NIT (supplemented with 2 mM KNO_3_) and allowed to acclimatize for 7 d at room temperature. Thereafter cells were passed once more into LBSW or LBSW-NIT and grown for 7 days. DNA was obtained as described above. For the anoxic data, microaerophilic breviate cultures were inoculated in 75 ml of LBSW-HEPES (2 mM HEPES, pH 8.0) or LBSW-NIT-HEPES in 100 ml serum flasks in three biological replicates. Unlike the microaerophilic conditions, we supplemented the anoxic cultures with prey bacteria (*Klebsiella pneumoniae,* 1 x 10^9^ cells/ml) because the prokaryotic community did not grow to sufficient density under anoxic conditions to maintain breviate growth. We supplemented with HEPES so as to best compare with the *Lenisia limosa* study[3]. Samples were incubated for 7 d at room temperature, and DNA was extracted from 250 μl of each microcosm. Because of these differences in experimental design, we do not compare the microaerophilic and anoxic data.

The V4 region of the 16S rRNA gene was amplified from each biological replicate in three technical replicates using the barcoded primers 515F[4] and 806R[5]. The gene was amplified with Phire Hot Start II DNA polymerase (ThermoScientific F122S, Lithuania), dNTP mix (VWR 733-1363, Denmark) and 2 μl of 1 ng/μl DNA, according to the protocol of the polymerase manufacturer. For details about primers and PCR conditions see Supplementary Datafile S2. Technical replicates were pooled together and purified with AMPure XP beads (Beckman Coulter A63880, Brea, California, USA), the DNA was eluted in 5 mM Tris-HCl pH 8.5. Library preparation and sequencing with MiSeq System (llumina) with paired-end (2 x 300 bp) were performed by Eurofins with their NGSelect Amplicon 2nd PCR service. Adapter sequence removal and read merging were performed by Eurofins using Cutadapt v2.7[6] and FLASH v2.2.00[7], respectively. Raw reads were deposited to NCBI, accession numbers can be found in Supplementary Datafile S3. The resulting data were processed using the qiime2 (v 2023.9) pipeline[8]. Reads were imported as as single end sequences, demultiplexed and denoised with the DADA2 pipeline, and reads were truncated at position 250[8, 9]. Reads that were identified in fewer than 3 samples or with a frequency lower than 500 were removed[8]. In addition we excluded the f__Enterobacteriaceae taxa corresponding to the food source that was added to the cultures. We explored the taxonomic composition of the samples using the pretrained SILVA 138 classifier[10, 11]. We used q2-gcn-norm plugin to normalize the data to copy number variation based on the rrnDB database (version 5.6) (https://github.com/Jiung-Wen/q2-gcn-norm). We converted the absolute abundance to relative abundance[8] and visualized the data in R. Differential abundance (Supplementary Datafile S3) across conditions was evaluated with ANCOM[12].

## Amplicon sequencing diversity analysis

ASV data were further analyzed in R (v4.2.2) for compositional analysis of relative abundance and visualization of alpha and beta diversity. Prior to calculating alpha diversity metrics, the data were rarefied to 40620 reads per sample. The alpha diversity metrics included Shannon diversity, which was used to calculate Pielou’s evenness as a complementary measure. Rarefaction and calculation of Shannon diversity were performed using the phyloseq package (v1.48.0). Statistical analysis of alpha diversity was performed to test for differences between conditions. Normality was tested with the Shapiro-Wilk test, and due to non-normal distributions, significant differences between conditions were identified using the Kruskal-Wallis test followed by Dunn’s test with Bonferroni correction (Supplementary datafile S3). Beta diversity was assessed using the Bray-Curtis dissimilarity metric, and Principal Coordinates Analysis (PCoA) was performed for visualization using the phyloseq package. Pairwise differences between conditions were tested using PERMANOVA via the pairwise.adonis2 function from the vegan package (v2.6.8). Statistical significance was determined at p ≤ 0.05, with R² values and F-statistics reported.

## Using DiSCo to predict dissimilatory sulfate reduction metabolism

To identify genes associated with dissimilatory sulfate reduction in the MAGs, DiSCo (version 1.0.0) was used, a Perl 5-based tool designed to automatically detect and classify proteins involved in Dsr-dependent dissimilatory sulfur metabolism (Supplementary Datafile S6). Predicted proteins were analyzed using DiSCo with a predefined Hidden Markov Model (HMM) library and an automated filtering step to retain high-confidence protein predictions[13].

## Detection of denitrification associated proteins

To distinguish putative nitric oxide reductases (NOR) from other heme-coper oxidases (HCO) we used previously reported HMMs[14] to classify the HCOs in the genomes using hmmsearch. Similarly, to distinguish nitrite reductase-related proteins NirK and NirS from other reductase proteins we queried our genomes using HMMs[15] with hmmsearch. The raw output of these analyses and the top scoring HMM with an evalue less than 1e-5 is summarized on figshare (doi.org/10.17044/scilifelab.28254575).

## Metagenomic sequencing, assembly and binning

Long-read metagenomic sequencing of PCE, LRM1b, LRM1N6, FB10N2 microcosm DNA was performed by the National Genomics Infrastructure Sweden with ONT ligation kit SQK-LSK109 on one ONT PromethION FLO-PRO002 flowcell. Demultiplexing, adaptor trimming and basecalling in super high accuracy was performed using Guppy 6.1.5 (dna_r9.4.1_450bps_hac_prom) . Sequencing of *Pygsuia biforma* DNA was performed in-house with ONT ligation kit SQK-NBD114.24 and sequenced on one ONT PromethION FLO-PRO114M flowcell. Demultiplexing, adaptator trimming and basecalling was performed in super high accuracy using Dorado 0.7.1 (r1041_e82_400bps_sup_v5.0.0). Reads were trimmed with chopper (-q 9 -l 500)<https://github.com/wdecoster/chopper>. DNA from each microcosm was assembled independently using Flye 2.9.1 (--meta)[16]. Quality was checked using metaquast 5.2[17]. Reads of each microcosm were mapped onto the corresponding assembly using minimap2 2.24-r1122[18] and samtools 1.14[19]. Contigs clustering, manual binning and gene calls were performed using anvi’o 8[20].

## Bacterial isolation and genome sequencing

In some cases, the genome bins of the metagenomes (*e.g., Desulfovibrionaceae* species from the *Pygsuia biforma* metagenome) were not of high quality. We therefore isolated the bacteria from the microcosms for whole genome sequencing (Fig. 3, tube icon). *Desulfovibrio glucosivorans* PB2 and *Pseudodesulfovibrio salinus* PB1 and LRM1 were isolated from the *P. biform*a microcosm, by diluting the culture and spreading it on SRBS (to 1 L: 2.32 g of Sulfate Reducing Broth Base Millipore 28228, India, 10 g of sodium thiosulfate, 4 ml of of 60% sodium DL-lactate solution Sigma-aldrich L4263, St. Louis, MO, USA, 33 g of instant ocean and 2% agar) and MBS (For 1 L: 37.4 g of Marine Broth BD Difco 2216, France, 4 ml of 60% sodium DL-lactate solution, and 2% agar. Adjust pH 7.2-7.5) agar plates, respectively. Plates were incubated in a glove box under an 80% N_2_, 10% CO_2_ and 10% H_2_ atmosphere at room temperature for around 7 d. To obtain pure cultures, single colonies were re-streaked in SRBS and MBS agar plates and subsequently grown in SRBS and MBS liquid media (without agar) for DNA purification. All the incubations were done in a glove box at room temperature. The DNA from *Desulfovibrio glucosivorans* PB2 was purified using Gene Jet kit for Gram negative bacteria (Thermo Scientific K0721, Lithuania), and further concentrated with AMPure XP beads according to the manufacturer’s instructions. The DNA from *Pseudodesulfovibrio salinus* PB1 was obtained with the phenol-chloroform method described above.

*Terasakiella halodenitrificans* LRM1 was isolated from the LRM1b microcosm, by diluting the culture and spreading it on marine broth (MB) plates supplemented with 2 mM KNO_3_. The inoculated plate was incubated in microaerophilic conditions (Anaerocult C, Millipore 1.32383.0001, Germany, in a BD BBL™ GasPak™ jar) for 96 h at room temperature. To obtain pure cultures, single colonies were re-streaked in MB and subsequently grown in MB liquid media for DNA purification. All the incubations were done in microaerophilic conditions. The DNA was purified with phenol-chloroform. DNA was quantified with Qubit dsDNA HS assay kit. DNA was sent to Eurofins for bacterial genome sequencing using Oxford Nanopore Technology. Bacterial genome assemblies and quality control of the assemblies were done by Eurofins: short and low-quality reads from the raw nanopore sequencing were removed using Filtlong v0.2.1 (https://github.com/rrwick/Filtlong). Bacterial genome de novo assemblies were done using Flye v2.9.3[21], and the resulting contigs were polished with Medaka v1.8 (https://github.com/nanoporetech/medaka). The quality of the assembled genomes were assessed using various tools, including QUAST v5.2[22], CheckM2 v1.0.1[23] and Mash v2.3[24]. To ensure the purity of the samples, the sequence-cleaded reads were mapped onto the assembly with minimap2 v2.24[18] and to call variations within the assembled genome Clair3 v1.0.4 was used[25].

## 16S rRNA phylogenetic trees

The 16S sequence from each bacterium of interest was extracted from the MAGs and from select type species from each of the major lineages under investigation were used as a query against the SILVA SINA search-and-classify[6] tool and retrieved 20 neighbours using a 90% cut-off value. These results were parsed into two datasets for each lineage: a dataset with only described bacteria and a dataset including environmental sequences. Each dataset was aligned using SINA to the global SSU alignment and gaps were removed using Wasabi default settings for each of the lineages of interest *Desulfovibrionaceae*, *Arcobacteraceae*, and *Terasakiella*. Phylogenies were inferred using IQTREE v2.0[26, 27] under the best scoring model of evolution decided by ModelFinder and 100 non-parametric bootstraps (-b 100) (Supplementary Datafile S7).

## Average Nucleotide Identity (ANI) comparisons between genomes

The taxonomic assignment of *Arcobacteraceae, Desulfovibrionaceae* and *Terasakiella* genomes was performed using GTDBtk 2.4.0 --denovo_wf[28]. To compare ANI between the new genomes, we used the EZBioCloud ANI calculator[29] (Supplementary Datafile S6). This allowed us to classify MAGs from different microcosms into the same species. For example *Halarcobacter azotofixans* PCE2 and *Halarcobacter azotofixans* FB1 (ANI of 99.49%), *Maridesulfovibrio spiralis* PCE5 and *Maridesulfovibrio spiralis* LRM2 (ANI of 99.46%), and *Halarcobacter ibericus* PCE3 and *Halarcobacter ibericus* 5MF (ANI of 94.66%). In addition, this method was used to compare *Desulfovibrio glucosivorans* PB2 with *Desulfovibrio glucosivorans* DMSS-1 (Reference genome: 2576861818) which is not present in the GTDB, but was investigated due to the high percent of identity of their 16S rRNA sequences (ANI of 97.61%).

## Detecting signs of co-evolution

To assess potential co-evolutionary patterns between breviate species and their associated bacterial communities inferred from the 16S amplicon sequencing data, a Mantel test was performed using the vegan R package (version 2.6.10). Bray–Curtis dissimilarities were calculated using the vegdist() function, based on copy number-normalized *Arcobacteraceae* abundances as well as on total bacterial domain-level data. Phylogenetic distances among breviate strains were computed as cophenetic distances from a maximum likelihood tree using the cophenetic.phylo() function in the ape R package (version 5.8.1). The resulting distance matrices were used as input for the Mantel test, which was run using the Pearson method with 999 permutations. To further assess whether phylogenetic congruence between breviates and potential symbionts exists, Procrustes Approach to Cophylogeny (PACo) was applied using the PACo R package (version 0.4.2). Phylogenetic distance matrices were calculated for both hosts and symbionts from a maximum likelihood tree using the cophenetic.phylo() function in the ape R package. A binary host–symbiont association matrix was constructed from co-occurrence data. These inputs were passed to the prepare_paco_data() function, followed by dimensionality reduction with add_pcoord(). The PACo analysis was performed using the PACo() function with 1,000 permutations and the Procrustes correlation (*R*²) as the goodness-of-fit metric[30]. The resulting fit and *P* value were used to assess the degree of congruence between host and symbiont phylogenies.

## Pseudogene detection

Annotated pseudogenes were identified from deposited NCBI GenBank files, except for Arcobacter EP1 and the *Arcobacter* MAG from *Osedax* which was laking NCBI annotation; Pseudofinder[31] was used instead. Each file was parsed using the Biopython library and genomic features annotated as CDS and marked with the /pseudo qualifier were extracted. For each pseudogene, we retrieved the associated locus tag (/locus_tag), the reason for pseudogenization (/note), any assigned Gene Ontology annotation (/GO_function) or the predicted product (/product). All results were aggregated into a single tab-separated output file for further analysis available in Supplementary Data S6.

## GapMind for prediction of amino acid biosynthesis

The predicted proteomes were downloaded directly from NCBI to keep the original annotation IDs, and were uploaded directly to the GapMind webpage for automated annotation of amino acid biosynthesis (<https://papers.genomics.lbl.gov/cgi-bin/gapView.cgi?set=aa>)[32, 33]. The results were summarized in Supplementary Datafile S6 and the raw results can be found in figshare (doi.org/10.17044/scilifelab.28254575). For comparison, the predicted proteomes of *Arcobacter* sp. EP1 (epibiont of *L. limosa*[3]), *Halarcobacter bivalvorium* LGM 26154 (NZ_CP031217.1), *Arcobacter nitrofigilis* DSM 7299 (NC_014166.1), *Malaciobacter marinus* JCM 15502 (NZ_CP032101.1) and the *Arcobacter* sp. epibiont of *Osedax* (GCA_029027265.1)[34] were downloaded and analyzed in GapMind. We were unable to find the predicted proteome of the *Arcobacter* epibiont of Osedax, we downloaded the genome and annotated with prokka 1.14.6[35] using galaxy 24.2.4.dev0[36].

## Fluorescence *in situ* hybridization

After fixation, the slides were rinsed in 2 × 50 mL distilled water for a total of 3 min and then air-dried. Slides were immersed in 50%, 80% and 100% ethanol for 3 min in each tube and air-dried. Dried cells on the slide were incubated with hybridization buffer (20 mM Tris-HCl, pH 7.6, 0.01% SDS, 900 mM NaCl), 5 ng/μ of FISH probe and an appropriate concentration of formamide (ThermoScientific 17899, Rockford, IL, USA, Supplementary Datafile S2), in a moist chamber at 46°C for 2 h[37, 38].

FISH probes were synthesized commercially (Supplementary Datafile S2), with identical fluorophores at the 5´- and 3´-end of the oligonucleotide[39]. After incubation, the slides were rinsed and incubated with 50 ml pre-warmed washing buffer (20 mM Tris-HCl, pH 7.5, 0.01% SDS, 5 mM EDTA and a suitable NaCl concentration depending on the %FA in the hybridization buffer, Supplementary Datafile S2) at 48°C for 30 min, and subsequently rinsed in distilled water for 40 s and air-dried[37, 38]. DAPI was added to each well with a final concentration of 2 μg/ml, slides were rinsed with distilled water and air-dried. Slides were mounted with SlowFade Diamond Antifade Mountant (ThermoScientific P36970, Eugene, Oregon, USA). We calculated the percentage of breviate-bacteria cells interacting by dividing the number of cells where the specific FISH-probe signal was detected surrounding or overlapping with the breviate cells, by the total number of breviate cells analyzed (percentaged and cells analyzed can be found Supplementary Datafile S2).

# SUPPLEMENTARY RESULTS & DISCUSSION

## All breviate associated bacteria encode genes for chemosensory and flagellar machinery

All the *Arcobacteraceae*, *Desulfovibrionaceae* and *Terasakiella* genomes described here encode for bacterial chemotaxis and flagellar proteins, suggesting these species might encode a chemosensory system. The pathway is composed of chemoreceptors, the histidine protein kinase chemotaxis protein (CheA) and two diffusible response regulators (CheY and CheB). CheY controls flagellar motor switching, whereas CheB controls chemoreceptor adaptation[40]. The encoded genes might be relevant for bacterial motility and invasion, and can contribute to syntrophic growth, as the flagella can facilitate chemotaxis to the syntrophic partner[3, 41]. There might be other genes and pathways besides the ones described here involved in the interactions with breviates, similar to the genes involved in colonization or virulence like the ones used by their animal-pathogenic relatives[42, 43].

## Limited evidence for co-diversification of bacteria and breviates in the microcosms

To investigate whether the composition of the *Arcobacteraceae* communities was shaped by breviate phylogeny, a Mantel test was performed, comparing Bray–Curtis dissimilarities (based on *Arcobacteraceae* abundances) with phylogenetic distances between breviate strains. The test did not detect a significant correlation (*r* = 0.25, *P* value = 0.3), suggesting that a phylogenetic signal in *Arcobacter* community composition could not be confirmed with the available data. A similar test using the complete bacterial community composition at the domain level also showed no significant correlation (*r* = 0.52, *P* value = 0.1). Additionally, a PACo analysis was performed to further assess congruence between breviates and bacteria. The analysis yielded a goodness-of-fit values based on the Procrustes correlation (*R*²) of 0.75 (*Arcobacteraceae*) and 0.73 (*Desulfovibrionaceae*), with a permutation-based *P* value of 0.279 (*Arcobacteraceae*) and 0.271 (*Desulfovibrionaceae*), indicating that the observed congruence is not statistically significant given the available data.

## Exploring amino acid biosynthetic capabilities and pseudogenization in breviate-associated bacteria

To determine if there were obvious gaps in biosynthetic capabilities in the breviate associated bacteria which could imply a dependence on the host, we predicted amino acid biosynthesis potential using GapMind[32, 33]. The *Arcobacteraceae* genomes lacked complete biosynthesis pathways for his, lys, chorismate, trp, phe and tyr (Supplementary Datafile S6 and doi.org/10.17044/scilifelab.28254575). Some of them, with the exception of *Halarcobacter ibericensis* PCE3, *Halarcobacter azotofixans* FB1 and *Arcobacter siniprincipis* 7KA lacked arg biosynthesis. *Halarcobacter ibericensis* PCE3, *Halarcobacter azotofixans* FB1, *Malaciobacter marinus* 2CH and 5JY, *Arcobacter denitrificans* LRM1 and *Arcobacter siniprincipis* 7KA lack the canonical ser biosynthesis pathway however, this represents a ‘known gap’ in the biosynthetic pathway. These predictions suggest that the breviate associated *Arcobacteraceae* species might be auxotrophic for some amino acids, and rely on other members of the community for their biosynthesis.This variability is not unusual; for example, *Lenisia limosa*-associated *Arcobacter* EP1 encode genes for the biosynthesis of all amino acid biosynthesis whereas the *Osedax*-associated *Arcobacter* does not encode complete pathways for at least seven amino acids. Other eukaryote-associated *Arcobacteraceae* (e.g, *Halarcobacter bivalvorium*[44], *Arcobacter nitrofigilis*[45] and *Malaciobacter marinus*[46]) show similar conservation patterns to our microcosm *Arcobacteraceae* (Supplementary Datafile S6). There is additionally high variability among the *Arcobacteraceae* as a whole; previous studies have showed varying degrees of completeness in lys, his and ser/thr biosynthesis across the *Arcobacteraceae*[47] The prediction of amino acid biosynthesis in *Terasakiella halodenitrificans* LRM1, suggested incomplete steps for met and trp biosynthesis with one enzyme missing in each pathway, metH and trpE, respectively. These predictions suggested that the bacterium is auxotroph for such amino acids.

The *Desulfovibrionaceae* genomes lack complete biosynthetic pathways for phe, ser and his. All the *Desulfovibrionaceae* genomes with the exception of *Desulfovibrio glucosivorans* PB2 have an incomplete pathway for trp biosynthesis. In the case of the his biosynthesis pathway, most of the genomes are missing only the hisN gene which represents a known gap in the pathway. Growth experiments in *Desulfovibrio vulgaris* showed that it did not require the addition of any amino acids for growth suggesting that there are yet-to-be discovered pathways for the biosynthesis of amino acids[48]. We therefore suspect that the present comparative genomics measures are insufficient to competely assess auxotrophy in these strains and further experimental validation is necessary.

We looked for predicted pseudogenes in NCBI annotations of our genomes as well as in reference *Arcobacteraceae* genomes. When we exclude the low-quality *Halarcobacter ibericensis* MAG, the breviate-associated bacteria have between 6-82 pseudogenes per genome represented by frameshifts and incomplete genes. Many of these predicted pseudogenes are annotated as transposases, however we could not observe a consistent pattern in the annotation of the pseudogenes. The true nature of these predicted pseudogenes is uncertain; although they may represent genuine gene remnants indicating genome reduction or functional degradation, we cannot exclude the possibility that some of them may be artifacts resulting from sequencing errors, assembly or annotation limitations. The complete genomes of the reference *Arcobacteraceae* strains range from 7-16 pseudogenes per genome. Further experimental validation or comparative genomic analysis would be required to confidently assess their functional status.

## Additional bacterial candidates that can interact with the breviates

Due to its high relative abundance in all the breviate microcosms, we have examined the *Fusibacter* bins (available doi.org/10.17044/scilifelab.28254575) and found that there are at least three different species across the microcosms. These bins do not appear to encode any of the nitrogen, sulfur, sulfate associated pathways of the discussed bacteria. The exception was a *Fusibacter* from the *P. biforma* microcosms that encoded for nitrogen fixation. *Fusibacter* has been previously reported to engage in an association with the anaerobic marine protist, *Carpediemononas frisia*, and was predicted to consume biomolecules such as amino acids, sugars and other low molecular weight organic acids that might be released directly by *C. frisia* or during the hydrolysis of proteins by other members of the community[49]. It is likely that the *Fusibacter* species present in the breviate microcosms share a similar metabolism, as it is common in other *Fusibacter* species[50]. A more detailed study of the *Fusibacter* genomes needs to be done in the future.

We also interrogated other bacterial members of the community and found that *Marinofilum* (*Ancylomarina*) present in PCE, FB10N2, LRM1b and LRM2N6 microcosms (also present in the *Carpediemononas frisia* consortium), encoded for complete DNRA. *Vibrionaceae* species from the different microcoms encode for DNRA. In addition, the *Celeribacter* species (*Rhodobacteracteaceae*) present in LRM1b and *Pygsuia biforma*, and *Marinobacterium* in FB10N2 encode for SOX pathways. These findings confirmed that the ecological role of the *Arcobacteraceae* species are not unique as multiple members of the community have similar roles. Whether *Fusibacter, Marinifilum* or other bacterial members in the microcosms interact with the breviates remains to be further explored. Nevertheless, they might be important players in the communities and required further investigation.

# SUPPLEMENTARY FIGURES & TABLES

| **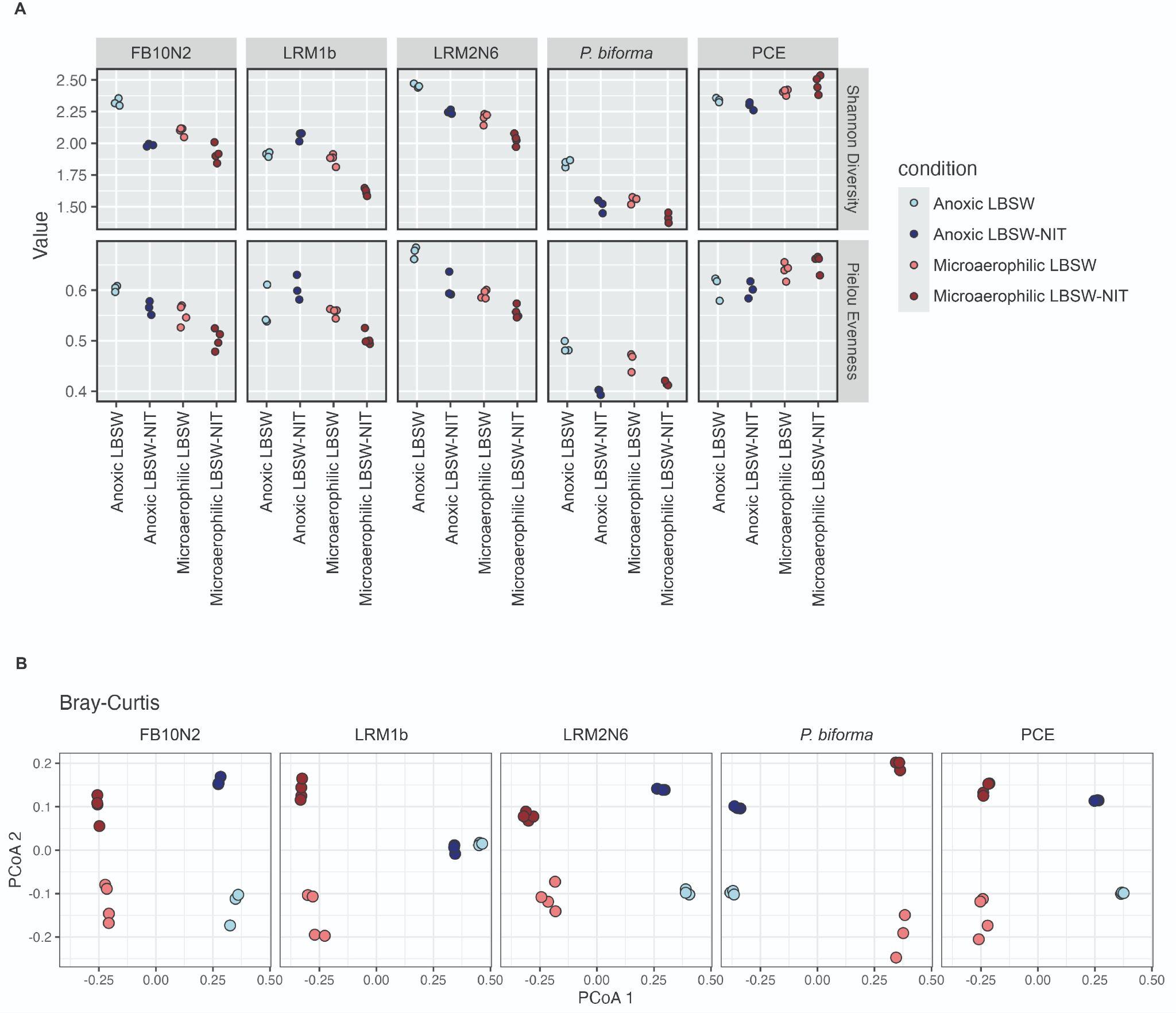** |
| --- |
| Supplementary figure S1: Differences in alpha and beta diversity of the bacterial community in the breviate microcosm. a) Alpha diversity plots of each breviate microcosm growing in anoxic or microaerophilic conditions, with or without nitrate, from left to right: FB10N2, LRM1b, LRM2N6, *P. biforma* and PCE. In the top, Shannon diversity, in the bottom, Pielou eveness. In terms of alpha diversity, Shannon Index was slightly higher in anoxic samples with sulfate for FB10N2, LRM2N6 and *P. biforma* whereas the Shannon Index was higher in anoxic samples supplemented with nitrate for LRM1b. For PCE, the Shannon Index was higher in microaerophilic samples with nitrate. In all the cases, the differences in Shannon Index is explained by the evenness, as observed with Pielou’s, and not by the richness of the species. b) Beta diversity PCoA plots of each breviate microcosm with labelling according to Figure S1a. The comparisons among communities were made using Bray-Curtis, the results indicated that all conditions (anoxic or microaerophilic, with or without nitrate) in all the samples differed significantly, with the exception of LRM1b anoxic samples with and without nitrate that clustered together in the PCoA plot, suggesting that the presence of sulfate in the anoxic sample is not changing the species. For statistical comparisons see Supplementary Datafile S3. |

| 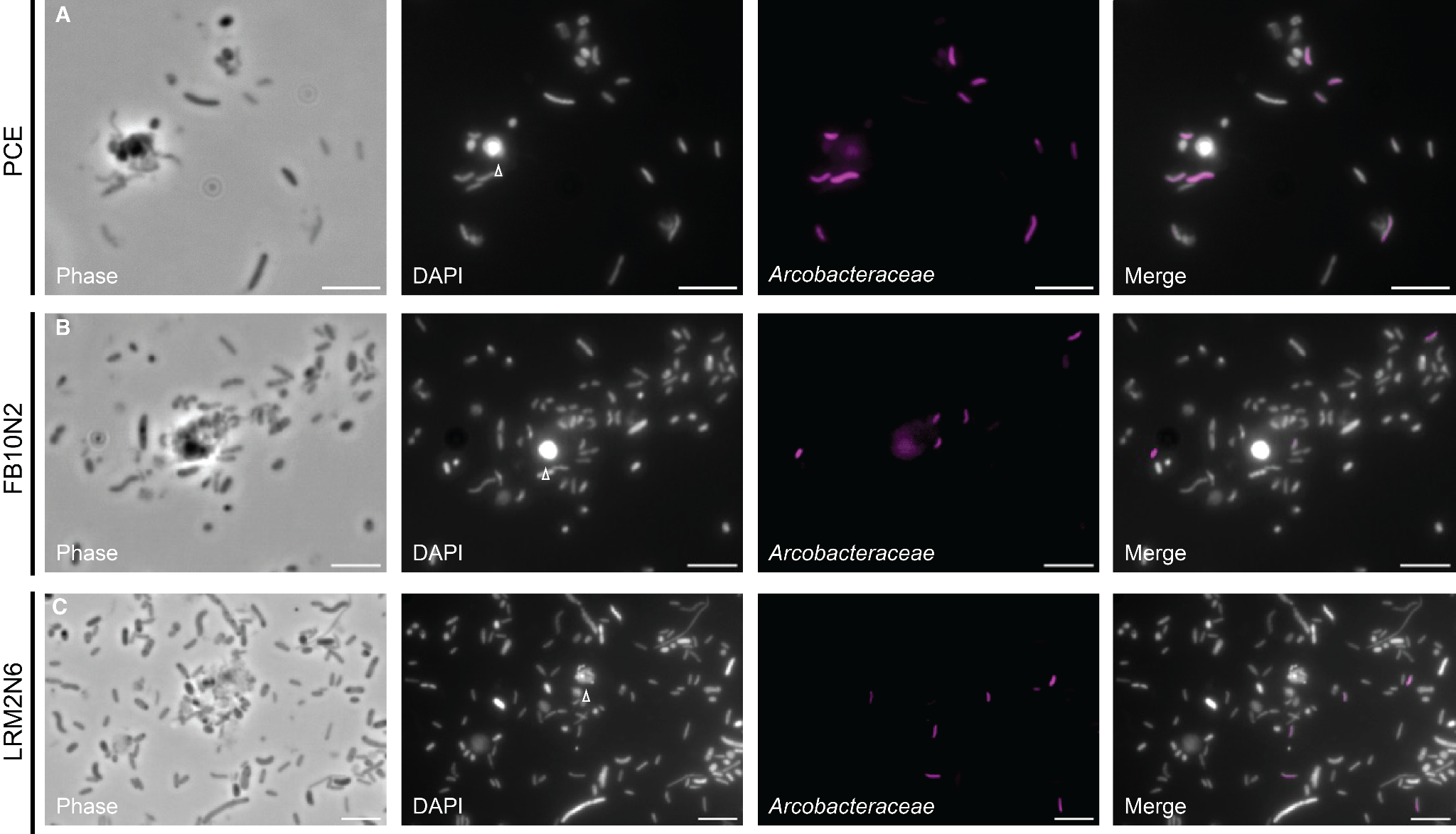 |
| --- |
| Supplementary figure S2: Fluorescence *in situ* hybridization (FISH) suggests a direct interaction of PCE, FB10N2 and LRM2N6 with *Arcobacteraceae*.   a) PCE, b) FB10N2 and c) LRM2N6 breviate microcosm were incubated on a slide overnight in anoxic conditions. Cultures were fixed (4% formaldehyde) and hybridized with 16S rRNA probes (20% formamide): Arc1430-Atto 488 and Arc94-Atto 488 targeting *Arcobacteraceae* cells, then stained with DAPI. Panels from left to right: Phase, DNA stained with DAPI (gray; arrowheads pointing to the breviate nuclei), *Arcobacteraceae* probes (magenta), and merge channel of DAPI with FISH probes. Scale bar 5 µm. |

| 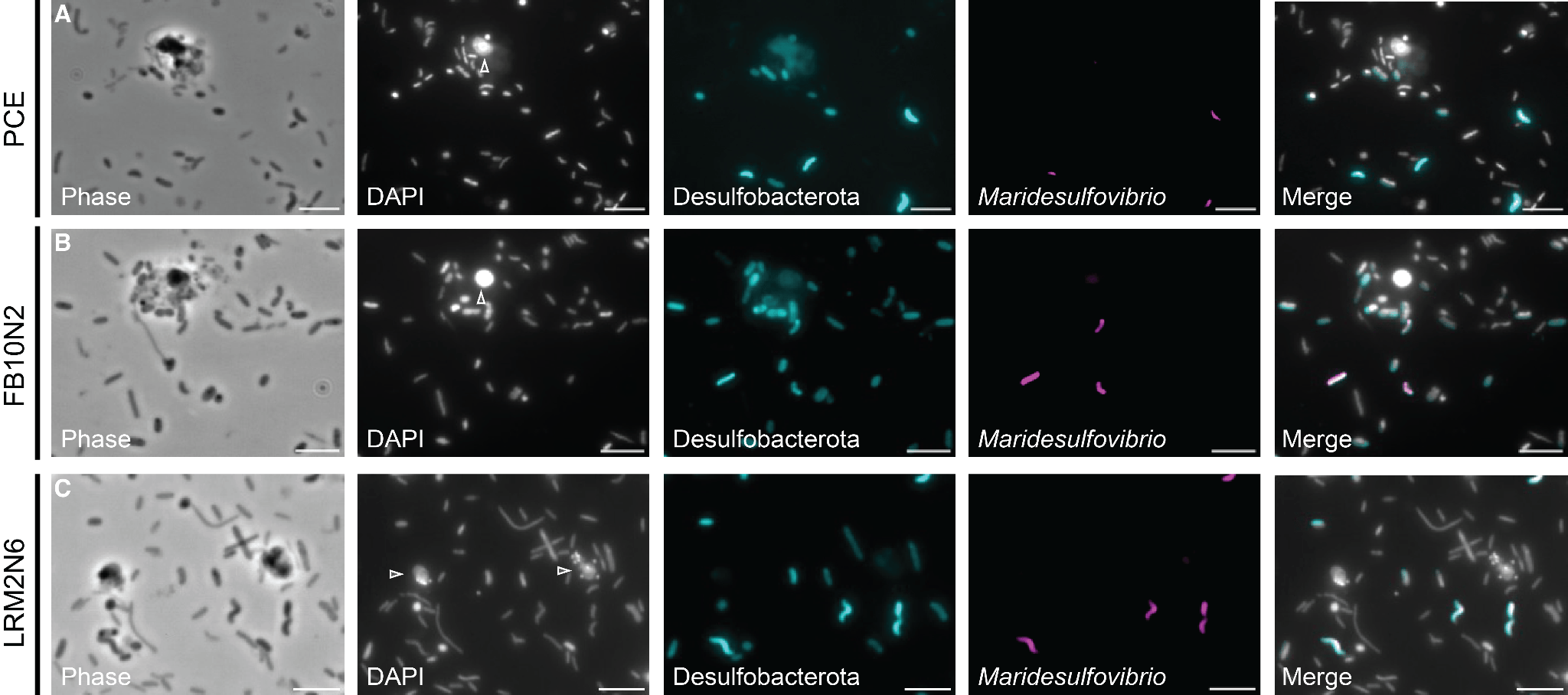 |
| --- |
| Supplementary figure S3: Fluorescence *in situ* hybridization (FISH) suggests a non-direct interaction of PCE, FB10N2 and LRM2N6 with *Maridesulfovibrio* species. a) PCE, b) FB10N2, and c) LRM2N6 breviate microcosm were incubated on a slide overnight in anoxic conditions. Cultures were fixed (4% formaldehyde) and hybridized with 16S rRNA probes (35% formamide): MD1 Atto 633 + MD2 Atto 633 targeting different *Maridesulfovibrio* species and delta495a Atto 550 targeting *Desulfobacterota*, then stained with DAPI. Panels from left to right: Phase, DNA stained with DAPI (gray; arrowheads pointing to the breviate nuclei), *Desulfobacterota* probes (cyan), *Maridesulfovibrio* probes (magenta), and merge channel of DAPI with FISH probes. Scale bar 5 µm. |

| 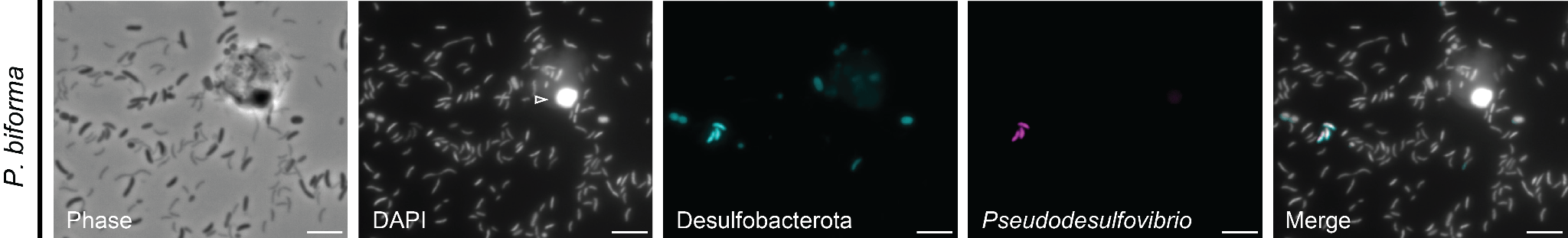 |
| --- |
| Supplementary figure S4: Fluorescence *in situ* hybridization (FISH) suggests a non-direct interaction of *P. biforma* with *Pseudodesulfovibrio*. *P. biforma* microcosms was incubated on a slide overnight in anoxic conditions. Cultures were fixed (4% formaldehyde) and hybridized with 16S rRNA probes (20% formamide): PD1-Atto 633 targeting *Pseudodesulfovibrio, and delta495a Atto 550 targeting Desulfobacterota,* then stained with DAPI. Panels from left to right: Phase, DNA stained with DAPI (gray; arrowheads pointing to the breviate nucleus), *Pseudodesulfovibrio* probe (magenta), and merge channel of DAPI with FISH probes. Scale bar 5 µm. |
|  |

# DESCRIPTION OF SUPPLEMENTARY DATA FILES

## Supplementary Datafile S1 (.xlsx)

This spreadsheet file includes multiple sheets with information about the sampling sites for the microcosms and the coordinates for the 18S sequences found in publicly available data.

## Supplementary Datafile S2 (.xlsx)

This spreadsheet contains multiple sheets summarizing the primer sequences, PCR conditions, phylogenetic tree information, FISH probes sequences and hybridization conditions, and estimation of percentages of bacterial-breviate cell interactions by FISH .

## Supplementary Datafile S3 (.xlsx)

This spreadsheet includes multiple sheets with information about the 16S amplicon analysis in the different breviate cultures grown with different conditions including the metadata file and accession numbers to the raw reads in NCBI. It also includes information about statistical analysis between the conditions (ANCOM) and diversity analysis .

## Supplementary Datafile S4 (.xlsx)

This spreadsheet shows the raw FlowCAM data for the breviate growth curve experiment in dSW (noE- no electron acceptor), dSW-NIT (nitrate) and dSW-SULF (sulfate) in anoxic conditions. Protists concentration was calculated at days 0, 3, 5, 7 and 10, using flowCAM.

## Supplementary Datafile S5 (.html)

This html file shows the workflow for calculating growth rates and statistical analysis of the FlowCAM cell count data.

## Supplementary Datafile S6 (.xlsx)

This spreadsheet contains multiple worksheets detailing the prokaryotic assembled genomes, Genbank accession numbers, the taxonomy classification using GTDBtk, average nucleotide identity comparisons between our assembled genomes and metabolic reconstruction, gapmind and pseudogene analysis.

## Supplementary Datafile S7 (.pdf)

This PDF shows the phylogenetic analysis of the 16S from breviate-associated bacteria from the *Arcobacteraceae*, *Desulfovibrionaceae,* and *Terasakiella* genomes. For *Arcobacteraceae* and *Desulfovibrionaceae* we performed an additional analysis of only cultured representatives. Breviate-associated sequences are coloured. Uncultured sequences from public databases are shown in grey.

## Supplementary Datafile S8 (.pdf)

This PDF shows the putative seqcode taxonomy of the breviate-associated bacteria.

## Figshare contents

- **16S_Breviate_Associated_Trees** - 16S rRNA phylogenetic trees for breviate-associated bacteria, including alignments, IQ-TREE outputs, and colorized tree visualizations.
- **18S_Breviate_Associated_Trees** - 18S rRNA sequences from multiple sources, alignments processed with SSU-align and SSU-mask, and phylogenetic trees generated using IQ-TREE.
- **Arcobacter_EP1_Annotation** - Gene prediction and annotation of *Arcobacter* sp. EP1 (GCA_001655195.1) using Prokka.
- **DIC_breviates** - Differential interference contrast (DIC) microscopy images of breviates.
- **FlowCam_classified_images** - classified FlowCam generated images, used for evaluating breviate concentrations.
- **FISH_breviate_microscosm** - Fluorescence *in situ* hybridization (FISH) images of breviate microcosms.
- **GTDBtk_Classification_and_Phylogeny** - GTDB-tk classification and phylogenetic placement of *Arcobacteraceae*, *Desulfovibrionaceae*, and *Terasakiella* genomes.
- **Metagenomic_MAGs** - Metagenome-assembled genomes (MAGs) generated through anvi’o binning and reassembled using Trycycler, with taxonomic classification, metabolic annotation, and quality assessment, along with associated scripts and logs
- **HCO_NirK_NirS –** hmmsearch output and summary of putative HCO, NirK and NirS genes

# REFERENCES

1. Medlin L et al. The characterization of enzymatically amplified eukaryotic 16S-like rRNA-coding regions. *Gene* 1988;**71**:491–499. https://doi.org/10.1016/0378-1119(88)90066-2

2. Sambrook J, Russell DW. Molecular cloning: a laboratory manual, 3rd ed. Cold Spring Harbor, N.Y: Cold Spring Harbor Laboratory Press, 2001.

3. Hamann E et al. Environmental Breviatea harbour mutualistic *Arcobacter* epibionts. *Nature* 2016;**534**:254–258. https://doi.org/10.1038/nature18297

4. Parada AE, Needham DM, Fuhrman JA. Every base matters: assessing small subunit rRNA primers for marine microbiomes with mock communities, time series and global field samples. *Environ Microbiol* 2016;**18**:1403–1414. https://doi.org/10.1111/1462-2920.13023

5. Apprill A et al. Minor revision to V4 region SSU rRNA 806R gene primer greatly increases detection of SAR11 bacterioplankton. *Aquat Microb Ecol* 2015;**75**:129–137. https://doi.org/10.3354/ame01753

6. Pruesse E, Peplies J, Glöckner FO. SINA: Accurate high-throughput multiple sequence alignment of ribosomal RNA genes. *Bioinformatics* 2012;**28**:1823–1829. https://doi.org/10.1093/bioinformatics/bts252

7. Magoč T, Salzberg SL. FLASH: fast length adjustment of short reads to improve genome assemblies. *Bioinformatics* 2011;**27**:2957–2963. https://doi.org/10.1093/bioinformatics/btr507

8. Bolyen E et al. Reproducible, interactive, scalable and extensible microbiome data science using QIIME 2. *Nat Biotechnol* 2019;**37**:852–857. https://doi.org/10.1038/s41587-019-0209-9

9. Callahan BJ et al. DADA2: High-resolution sample inference from Illumina amplicon data. *Nat Methods* 2016;**13**:581–583. https://doi.org/10.1038/nmeth.3869

10. Robeson MS et al. RESCRIPt: Reproducible sequence taxonomy reference database management for the masses. Bioinformatics, 2020.

11. Bokulich NA et al. Optimizing taxonomic classification of marker-gene amplicon sequences with QIIME 2’s q2-feature-classifier plugin. *Microbiome* 2018;**6**:90. https://doi.org/10.1186/s40168-018-0470-z

12. Mandal S et al. Analysis of composition of microbiomes: a novel method for studying microbial composition. *Microb Ecol Health Dis* 2015;**26**. https://doi.org/10.3402/mehd.v26.27663

13. Neukirchen S, Sousa FL. DiSCo: a sequence-based type-specific predictor of Dsr-dependent dissimilatory sulphur metabolism in microbial data. *Microb Genomics* 2021;**7**. https://doi.org/10.1099/mgen.0.000603

14. Murali R, Hemp J, Gennis RB. Evolution of quinol oxidation within the heme‑copper oxidoreductase superfamily. *Biochim Biophys Acta BBA - Bioenerg* 2022;**1863**:148907. https://doi.org/10.1016/j.bbabio.2022.148907

15. Pold G et al. Phylogenetics and environmental distribution of nitric oxide-forming nitrite reductases reveal their distinct functional and ecological roles. *ISME Commun* 2024;**4**:ycae020. https://doi.org/10.1093/ismeco/ycae020

16. Kolmogorov M et al. metaFlye: scalable long-read metagenome assembly using repeat graphs. *Nat Methods* 2020;**17**:1103–1110. https://doi.org/10.1038/s41592-020-00971-x

17. Mikheenko A, Saveliev V, Gurevich A. MetaQUAST: evaluation of metagenome assemblies. *Bioinformatics* 2016;**32**:1088–1090. https://doi.org/10.1093/bioinformatics/btv697

18. Li H. Minimap2: pairwise alignment for nucleotide sequences. *Bioinformatics* 2018;**34**:3094–3100. https://doi.org/10.1093/bioinformatics/bty191

19. Danecek P et al. Twelve years of SAMtools and BCFtools. *GigaScience* 2021;**10**:giab008. https://doi.org/10.1093/gigascience/giab008

20. Eren AM et al. Community-led, integrated, reproducible multi-omics with anvi’o. *Nat Microbiol* 2021;**6**:3–6. https://doi.org/10.1038/s41564-020-00834-3

21. Kolmogorov M et al. Assembly of long, error-prone reads using repeat graphs. *Nat Biotechnol* 2019;**37**:540–546. https://doi.org/10.1038/s41587-019-0072-8

22. Gurevich A et al. QUAST: quality assessment tool for genome assemblies. *Bioinformatics* 2013;**29**:1072–1075. https://doi.org/10.1093/bioinformatics/btt086

23. Chklovski A et al. CheckM2: a rapid, scalable and accurate tool for assessing microbial genome quality using machine learning. *Nat Methods* 2023;**20**:1203–1212. https://doi.org/10.1038/s41592-023-01940-w

24. Ondov BD et al. Mash: fast genome and metagenome distance estimation using MinHash. *Genome Biol* 2016;**17**:132. https://doi.org/10.1186/s13059-016-0997-x

25. Zheng Z et al. Symphonizing pileup and full-alignment for deep learning-based long-read variant calling. *Nat Comput Sci* 2022;**2**:797–803. https://doi.org/10.1038/s43588-022-00387-x

26. Minh BQ et al. IQ-TREE 2: New models and efficient methods for phylogenetic inference in the genomic era. *Mol Biol Evol* 2020;**37**:1530–1534. https://doi.org/10.1093/molbev/msaa015

27. Kalyaanamoorthy S et al. ModelFinder: fast model selection for accurate phylogenetic estimates. *Nat Methods* 2017;**14**:587–589. https://doi.org/10.1038/nmeth.4285

28. Chaumeil P-A et al. GTDB-Tk: a toolkit to classify genomes with the Genome Taxonomy Database. *Bioinformatics* 2020;**36**:1925–1927. https://doi.org/10.1093/bioinformatics/btz848

29. Yoon S-H et al. A large-scale evaluation of algorithms to calculate average nucleotide identity. *Antonie Van Leeuwenhoek* 2017;**110**:1281–1286. https://doi.org/10.1007/s10482-017-0844-4

30. Balbuena JA, Míguez-Lozano R, Blasco-Costa I. PACo: A Novel Procrustes Application to Cophylogenetic Analysis. *PLoS ONE* 2013;**8**:e61048. https://doi.org/10.1371/journal.pone.0061048

31. Syberg-Olsen MJ et al. Pseudofinder: Detection of Pseudogenes in Prokaryotic Genomes. *Mol Biol Evol* 2022;**39**:msac153. https://doi.org/10.1093/molbev/msac153

32. Price MN, Deutschbauer AM, Arkin AP. GapMind: Automated Annotation of Amino Acid Biosynthesis. *mSystems* 2020;**5**:e00291-20. https://doi.org/10.1128/mSystems.00291-20

33. Price MN et al. Improving the annotation of amino acid biosynthesis pathways: GapMind 2024. 2024. Microbiology, 2024.

34. Goffredi SK et al. A dynamic epibiont community associated with the bone-eating polychaete genus *Osedax*. *mBio* 2023;e03140-22. https://doi.org/10.1128/mbio.03140-22

35. Seemann T. Prokka: rapid prokaryotic genome annotation. *Bioinformatics* 2014;**30**:2068–2069. https://doi.org/10.1093/bioinformatics/btu153

36. The Galaxy Community et al. The Galaxy platform for accessible, reproducible, and collaborative data analyses: 2024 update. *Nucleic Acids Res* 2024;**52**:W83–W94. https://doi.org/10.1093/nar/gkae410

37. Jerlström-Hultqvist J et al. A unique symbiosome in an anaerobic single-celled eukaryote. *Nat Commun* 2024;**15**:9726. https://doi.org/10.1038/s41467-024-54102-7

38. Bridger JM, Volpi EV (eds). Fluorescence in situ Hybridization (FISH). Totowa, NJ: Humana Press, 2010.

39. Behnam F et al. A Straightforward DOPE (Double Labeling of Oligonucleotide Probes)-FISH (Fluorescence *In Situ* Hybridization) Method for Simultaneous Multicolor Detection of Six Microbial Populations. *Appl Environ Microbiol* 2012;**78**:5138–5142. https://doi.org/10.1128/AEM.00977-12

40. Sim MS et al. Effect of electron donors on the fractionation of sulfur isotopes by a marine Desulfovibrio sp. *Geochim Cosmochim Acta* 2011;**75**:4244–4259. https://doi.org/10.1016/j.gca.2011.05.021

41. Krumholz LR et al. Syntrophic Growth of *Desulfovibrio alaskensis* Requires Genes for H_2_ and Formate Metabolism as Well as Those for Flagellum and Biofilm Formation. *Appl Environ Microbiol* 2015;**81**:2339–2348. https://doi.org/10.1128/AEM.03358-14

42. Baztarrika I et al. Foodborne and waterborne *Arcobacter* species exhibit a high virulent activity in Caco-2. *Food Microbiol* 2024;**118**:104424. https://doi.org/10.1016/j.fm.2023.104424

43. Xie R et al. *Desulfovibrio vulgaris* interacts with novel gut epithelial immune receptor LRRC19 and exacerbates colitis. *Microbiome* 2024;**12**:4. https://doi.org/10.1186/s40168-023-01722-8

44. Levican A et al. *Arcobacter bivalviorum* sp. nov. and *Arcobacter venerupis* sp. nov., new species isolated from shellfish. *Syst Appl Microbiol* 2012;**35**:133–138. https://doi.org/10.1016/j.syapm.2012.01.002

45. McCLUNG CR, Patriquin DG, Davis RE. *Campylobacter nitrofigilis* sp. nov., a Nitrogen-Fixing Bacterium Associated with Roots of *Spartina alterniflora* Loisel. *Int J Syst Bacteriol* 1983;**33**:605–612. https://doi.org/10.1099/00207713-33-3-605

46. Kim HM, Hwang CY, Cho BC. *Arcobacter marinus* sp. nov. *Int J Syst Evol Microbiol* 2010;**60**:531–536. https://doi.org/10.1099/ijs.0.007740-0

47. Buzzanca D et al. *Arcobacteraceae* comparative genome analysis demonstrates genome heterogeneity and reduction in species isolated from animals and associated with human illness. *Heliyon* 2023;**9**:e17652. https://doi.org/10.1016/j.heliyon.2023.e17652

48. Price MN et al. Filling gaps in bacterial amino acid biosynthesis pathways with high-throughput genetics. *PLOS Genet* 2018;**14**:e1007147. https://doi.org/10.1371/journal.pgen.1007147

49. Hamann E et al. Syntrophic linkage between predatory *Carpediemonas* and specific prokaryotic populations. *ISME J* 2017;**11**:1205–1217. https://doi.org/10.1038/ismej.2016.197

50. Acosta-Grinok M et al. Looking for the mechanism of arsenate respiration of *Fusibacter* sp. strain 3D3, independent of ArrAB. *Front Microbiol* 2022;**13**:1029886. https://doi.org/10.3389/fmicb.2022.1029886
