# Supplementary material for: Anaerobic breviate protist survival in microcosms depends on microbiome metabolic function
Source: ISME J. 2025 Aug 8;19(1):wraf171. doi: 10.1093/ismejo/wraf171 (PMC12453579; doi:10.1093/ismejo/wraf171)
Supplement: Supplementary_DataFileS8_seqcode_wraf171 [file supplementary_datafiles8_seqcode_wraf171.pdf]

Register list for 11 new names of bacteria associated with protists

Submitted by Courtney, Stairs

Genus *Thalassodesulfovibrio*

Etymology
[tha.las.so.de.sul.fo.vi'bri.o] **Gr. fem. n.** *thalassa*, sea; **N.L. masc. n.** *Desulfovibrio*, the bacterial genus referring to a vibrio that reduces sulfur compounds; **N.L. masc. n.** *Thalassodesulfovibrio*, a marine bacterium capable of sulfur reduction

Nomenclatural type
Species *Thalassodesulfovibrio aquaticus*[Ts]

Description
The species is established on the basis of MiGA taxonomic novelty analysis, and the type material is the genome CP182354

Classification
Bacteria » *Desulfobacterota* » *Desulfovibrionia* » *Desulfovibrionales* » *Desulfovibrionaceae* » *Thalassodesulfovibrio*

Registry URL
https://seqco.de/i:49743

Species *Marinarcus sediminis*

Etymology
[se.di.mi'nis] **L. gen. n.** *sediminis*, of sediment

Nomenclatural type
INSDC Nucleotide: CP182357 [Ts]

Description
The species is established on the basis of MiGA taxonomic novelty analysis, and the type material is the genome CP182357

Classification
Bacteria » *Pseudomonadota* » *Epsilonproteobacteria* » *Campylobacterales* » *Arcobacteraceae* » *Marinarcus* » *Marinarcus sediminis*

Registry URL
https://seqco.de/i:49739

Species *Thalassodesulfovibrio aquaticus*[Ts]

Etymology
[a.qua'ti.cus] **L. masc. adj.** *aquaticus*, living, growing, or found in or by the water, aquatic

Nomenclatural type
INSDC Nucleotide: CP182354 [Ts]

Description
The species is established on the basis of MiGA taxonomic novelty analysis, and the type material is the genome CP182354

Classification
Bacteria » *Desulfobacterota* » *Desulfovibrionia* » *Desulfovibrionales* » *Desulfovibrionaceae* » *Thalassodesulfovibrio* » *Thalassodesulfovibrio aquaticus*[Ts]

Registry URL
https://seqco.de/i:49749

Species *Arcobacter denitrificans*

Etymology
[de.ni.tri.fi'cans] **N.L. masc. part. adj.** *denitrificans*, denitrifying

Nomenclatural type
INSDC Nucleotide: CP182350 [Ts]

Description
The species is established on the basis of MiGA taxonomic novelty analysis, and the type material is the genome CP182350

Classification
Bacteria » *Pseudomonadota* » *Epsilonproteobacteria* » *Campylobacterales* » *Arcobacteraceae* » *Arcobacter* » *Arcobacter denitrificans*

Registry URL
https://seqco.de/i:49746

Species *Maridesulfovibrio pacificus*

Etymology
[pa.ci'fi.cus] **N.L. masc. adj.** *pacificus*, pacific, pertaining to the Pacific Ocean, the source of the genome

Nomenclatural type
NCBI Assembly: GCA_048612815.1 [Ts]

Reference Strain
Strain scJ0038867: JBLZNE000000000

Description
The species is established on the basis of MiGA taxonomic novelty analysis, and the type material is the genome JBLZNE000000000

Classification
Bacteria » *Desulfobacterota* » *Desulfovibrionia* » *Desulfovibrionales* » *Desulfovibrionaceae* » *Maridesulfovibrio* » *Maridesulfovibrio pacificus*

Registry URL
https://seqco.de/i:49841

Species *Arcobacter siniprincipis*

Etymology
[si.ni.prin'ci.pis] **L. masc. n.** *sinus*, bay or cove; **L. masc. n.** *princeps*, prince; **L. masc. gen. n.** *siniprincipis*, from Prince Cove

Nomenclatural type
INSDC Nucleotide: CP182343 Ts

Description
The species is established on the basis of MiGA taxonomic novelty analysis, and the type material is the genome CP182343

Classification
Bacteria » *Pseudomonadota* » *Epsilonproteobacteria* » *Campylobacterales* » *Arcobacteraceae* » *Arcobacter* » *Arcobacter siniprincipis*

Registry URL
https://seqco.de/i:49748

Species *Maridesulfovibrio spiralis*

Etymology
[spi.ra'lis] **L. masc. adj.** *spiralis*, spiraling

Nomenclatural type
INSDC Nucleotide: CP182344 Ts

Description
The species is established on the basis of MiGA taxonomic novelty analysis, and the type material is the genome CP182344

Classification
Bacteria » *Desulfobacterota* » *Desulfovibrionia* » *Desulfovibrionales* » *Desulfovibrionaceae* » *Maridesulfovibrio* » *Maridesulfovibrio spiralis*

Registry URL
https://seqco.de/i:49744

Species *Terasakiella halodenitrificans*

Etymology
[ha.lo.de.ni.tri.fi'cans] **Gr. masc. n.** *hals*, salt; **N.L. pres. part.** *denitrificans*, denitrifying; **N.L. fem. part. adj.** *halodenitrificans*, denitrifying bacterium that is tolerant to salty environments

Nomenclatural type
INSDC Nucleotide: CP182348 Ts

Description
The species is established on the basis of MiGA taxonomic novelty analysis, and the type material is the genome CP182348

Classification
Bacteria » *Pseudomonadota* » *Alphaproteobacteria* » *Rhodospirillales* » *Terasakiellaceae* » *Terasakiella* » *Terasakiella halodenitrificans*

Registry URL
https://seqco.de/i:49747

Species *Halarcobacter azotofixans*

Etymology
[a.zot.o.fix'ans] **N.L. neut. n.** *azotum*, nitrogen; **N.L. pres. part.** *fixans*, fixing; **N.L. masc. part. adj.** *azotofixans*, nitrogen-fixing

Nomenclatural type
INSDC Nucleotide: CP182352 Ts

Description
The species is established on the basis of MiGA taxonomic novelty analysis, and the type material is the genome CP182352

Classification
Bacteria » *Pseudomonadota* » *Epsilonproteobacteria* » *Campylobacterales* » *Arcobacteraceae* » *Halarcobacter* » *Halarcobacter azotofixans*

Registry URL
https://seqco.de/i:49741

Species *Halarcobacter ibericensis*

Etymology
[i.be.ri.cen'sis] **N.L. masc. adj.** *ibericensis*, from the Iberian Peninsula

Nomenclatural type
INSDC Nucleotide: CP187315 Ts

Description
The species is established on the basis of MiGA taxonomic novelty analysis, and the type material is the genome CP182347

Classification
Bacteria » *Pseudomonadota* » *Epsilonproteobacteria* » *Campylobacterales* » *Arcobacteraceae* » *Halarcobacter* » *Halarcobacter ibericensis*

Registry URL
https://seqco.de/i:49742

Species *Pseudodesulfovibrio salinus*

Etymology
[sa.li'nus] **L. masc. adj.** *salinus*, of salt, referring to this taxon's recovery from the sea

Nomenclatural type
INSDC Nucleotide: CP182342 Ts

Description
The species is established on the basis of MiGA taxonomic novelty analysis, and the type material is the genome CP182342

Classification
Bacteria » *Desulfobacterota* » *Desulfovibrionia* » *Desulfovibrionales* » *Desulfovibrionaceae* » *Pseudodesulfovibrio* » *Pseudodesulfovibrio salinus*

Registry URL
https://seqco.de/i:49745
